# Aggregation Limiting Cell-Penetrating Peptides Derived from Protein Signal Sequences

**DOI:** 10.3390/ijms24054277

**Published:** 2023-02-21

**Authors:** Ly Porosk, Heleri Heike Härk, Renata Naporano Bicev, Ilja Gaidutšik, Jekaterina Nebogatova, Eger-Jasper Armolik, Piret Arukuusk, Emerson Rodrigo da Silva, Ülo Langel

**Affiliations:** 1 Institute of Technology, University of Tartu, Nooruse 1, 50411 Tartu, Estonia; 2 Departamento de Biofísica, Universidade Federal de São Paulo, São Paulo 04023-062, Brazil; 3 Department Biochemistry and Biophysics, Stockholm University, S.Arrheniusv. 16B, Room C472, 106 91 Stockholm, Sweden

**Keywords:** amyloid beta, cell-penetrating peptides, Alzheimer’s disease, aggregation, peptides

## Abstract

Alzheimer’s disease (AD) is the most common neurodegenerative disease (ND) and the leading cause of dementia. It is characterized by non-linear, genetic-driven pathophysiological dynamics with high heterogeneity in the biological alterations and the causes of the disease. One of the hallmarks of the AD is the progression of plaques of aggregated amyloid-β (Aβ) or neurofibrillary tangles of Tau. Currently there is no efficient treatment for the AD. Nevertheless, several breakthroughs in revealing the mechanisms behind progression of the AD have led to the discovery of possible therapeutic targets. Some of these include the reduction in inflammation in the brain, and, although highly debated, limiting of the aggregation of the Aβ. In this work we show that similarly to the Neural cell adhesion molecule 1 (NCAM1) signal sequence, other Aβ interacting protein sequences, especially derived from Transthyretin, can be used successfully to reduce or target the amyloid aggregation/aggregates in vitro. The modified signal peptides with cell-penetrating properties reduce the Aβ aggregation and are predicted to have anti-inflammatory properties. Furthermore, we show that by expressing the Aβ-EGFP fusion protein, we can efficiently assess the potential for reduction in aggregation, and the CPP properties of peptides in mammalian cells.

## 1. Introduction

Amyloidosis is as a group of diseases characterized by the extracellular or intracellular deposition of insoluble amyloid fibrils. These aggregates form from the proteins which are normally soluble. Due to conformational changes caused by diverse mechanisms these proteins may form aggregates. The AD is one of the leading fatal NDs in the developed countries where the average life expectancy of the population is increasing. AD significantly reduces the life quality of the patient and leads to their premature death.

The main component of the extracellular amyloid plaque is the Aβ, which has a significant role in the progression of the AD [1]. The Aβ appears and the Aβ aggregates progressively accumulate outside the neurons years before the plaques form. The plaques are associated with the advanced and irreversible characteristic neurocognitive decline in the AD [2]. The Aβ causes the formation of hyper-phosphorylated tau protein neurofibrillary tangles inside the neurons, eventually leading to the loss of neurons [3]. The Aβ is a 39–43 amino acid residue peptide generated upon sequential and alternate cleavages of the amyloid precursor protein. These cleavages result in the generation of two types of peptide isoforms Aβ_1–40_ (Aβ_40_) and Aβ_1–42_ (Aβ_42_), also found in the normal physiology. The imbalance between the production and the clearance of Aβ is considered as a possible cause of the amyloid deposition in the AD brains. The Aβ peptides can form heterogeneous oligomers and fibrils under physiological conditions. The oligomers and diffusible Aβ, such as proto-fibrils, pre-fibrillary aggregates, and Aβ-derived diffusible ligands are considered the components leading the toxicity [4,5,6]. It has been debated that in the AD the pathogenesis is not directly related to the plaque burden. As suggested by a recent study, the combination therapies addressing both the amyloid plaque build-up and the neuro-inflammation may prove effective in the treatment and prevention of AD [7].

Peptide-based molecules are a class of possible therapeutic and diagnostic tools for many diseases, including the AD. The higher efficacies and fewer associated side effects give peptide-based drugs an advantage over small molecule therapeutics [8]. For example, the ACI-80 peptide derivates which bind the Aβ are suitable for in vivo imaging and are possibly useful for early diagnosis and therefore a better prognosis for the AD patients [8]. Whereas effective binder, it is unable to diffuse the plaques. The inhibition of the aggregation of the Aβ in order to limit the progression of AD has emerged as one of the promising approaches/targets, although its clinical output is still debated. Among others, the majority of peptide-based inhibitors for the Aβ aggregation are peptide fragments designed to bind to the Aβ regions that are critical for their aggregation. The peptides target the Aβ fibril formation and elongation, leading to the inhibition of the monomer/oligomer formations. The reduction in the rate of aggregation or reduction in formation of aggregates accompanied with maintenance of none to low inflammation levels may be a useful strategy to prevent the onset and the progression of clinical symptoms in the patients.

Peptide-based mimetics have emerged as a promising tool to mimic protein-protein interactions. The affinity and the selectivity of the peptides can be significantly higher, when compared to the small-molecule therapeutics, and the efficacies and cost-effectiveness higher than that for the protein-based therapeutics. A variety of small peptides derived from the proteins that inhibit the aggregation of the Aβ and reduce its toxic effects were described in several works [8,9,10,11]. For example, peptides derived from the protein transthyretin (TTR) that mimic the Aβ-binding domains of TTR. The TTR is an evolutionarily conserved serum and cerebrospinal fluid (CSF) protein. It is a homo-tetrameric protein, and its each monomer contains two four-stranded β-sheets. The TTR is known to directly bind soluble, oligomeric, and fibrils of the Aβ, playing a role in the clearance of Aβ. When compared to the human protein, the mouse TTR binds to Aβ_42_ oligomers more efficiently [12], and is more stable. TTR co-localizes with Aβ in plaques, and probably binds primary and secondary nucleation of Aβ and by this reduces its toxicity to cells [13]. It has been shown that the dissociation of the tetrameric TTR may also cause transthyretin amyloidosis, and the dissociation of the TTR tetramer, required for its amyloid pathogenesis, may be necessary to prevent cellular toxicity from the Aβ oligomers [14]. The TTR_102–117_ and TTR_74–83_ derived peptides were developed for this purpose [15]. Its further design into a cyclic peptide cG8 [16] showed a reduction potential for limiting the aggregation of the Aβ.

Aside from the TTR, the peptides derived from the Apolipoprotein E (ApoE) sequence, ApoE_133–149_ may exhibit protection from the Aβ toxicity [17], although the mechanism is not known. In another work, it was shown that the ApoE mimetic peptide COG112 has a protective effect against the AD [18]. The protein Apolipoprotein E (ApoE) and its polymorphisms are linked to the pathogenesis of the late-onset and the sporadic AD [19]. ApoE facilitates the deposition and fibrillogenesis of the Aβ, and may participate in the clearance of the Aβ. The lipoprotein receptor related to the protein binding segment of ApoE linked to 16 lysines has been shown to deliver cargo across the blood-brain barrier (BBB) [20,21]. Related to the ApoE, the complex formed between ApoE and Apolipoprotein AII (ApoAII) bind to the Aβ much more strongly than does the monomeric ApoE, probably leading to the increased clearance of the Aβ. The addition of the ApoAII maintained higher cellular viability than just ApoE, probably due to formation of ApoE-AII complex and increased binding to the Aβ [19].

Over the years several other proteins have been linked to the onset, the progression, or the lower risk of developing the AD. The involvement of the TTR, Apolipoproteins, and the Cystatin C (CysC) in the interactions with the Aβ_42_ has been thoroughly revised [22]. Lysozymes (LYZ) are a part of the innate immune system. They have primarily a bacteriolytic function, and the lysozymes in the tissues and the body fluids are associated with the monocyte-macrophage system and enhance the activity of immune-agents. LYZ is also present in CSF, and their level increases especially during inflammation, but also due to different diseases [23]. In patients with the AD the increased LYZ levels are triggered by the Aβ_42_. The LYZ binds to the Aβ_40_ and the Aβ_42_ during the early stages of fibrillation, and through this prevents their toxic effects [24,25]. Co-expression of Aβ_42_ and LYZ have been shown to reduce the Aβ-led toxicity [24]. Because of its ability to self-assemble, lysozyme is often used as a model molecule to study protein stability, folding, and aggregation [26]. The CysC is localized in the neurons and glia. Its expression is increased in response to injury and it has been shown to co-localize in the brain with amyloid deposits in patients with the AD, in senile plaque, and in the walls of blood vessel. Contradictory conclusions were reached from multiple studies, suggesting that increased CysC cellular expression in the brain is either associated with the ND process, or alternatively is a part of a neuroprotective response aimed at prevention of neurodegeneration. It was suggested that reduced secretion of CysC into extracellular body fluids can hamper the ability of the brain to prevent ND in various pathological conditions [27,28]. The interactions and reduction-potential of the CysC have been linked mainly with human CysC protein.

The scavenger receptors are expressed on the surface of microglia, and act as receptors for the Aβ [29]. In adults the Scavenger receptor class B is only expressed on the astrocytes. In a mouse model of the AD, the reduction in the expression of the Scavenger receptor class B member 1 (SCARB-1) protein increased the deposition of the Aβ plaque, and had no effect on the microglial accumulation around the Aβ plaques and in fact worsened the cognitive defects in learning and memory [30]. The SCARB1 has been identified on the astrocytes and the vascular smooth muscle cells in the AD brain and has been shown to mediate the adhesion of microglia to the fibrillary amyloid-β (Aβ) [30]. The Neuronal acetylcholine receptor subunit alpha-7 (ACHA) and the Transforming growth factor beta-2 proprotein (TGFB) proteins have been shown to increase the aggregation of Aβ and the onset of AD [31]. The ACHA is a homopentameric ligand-gate ion channel with five acetylcholine binding sites, found in the post-synaptic cell membrane. It has been suggested that the ACHA may be a critical link between the inflammation and the neurodegeneration in the AD. Several studies indicate that ACHA mediates the toxicity of the Aβ by direct binding [32] and acts as a triggering factor of the neuronal cell death, the AD pathology, and the related inflammation [33,34]. The TGFP is an extracellularly found protein that is involved in a broad range of biological functions, such as cell death, inflammation, immunological reactions, etc.

In a cell-penetrating peptide (CPP) related work, it was shown that the cellular prion protein (PrP) could interact with the Aβ [35]. Furthermore, the synthetic peptide derived from the PrP was effective against prion, and it binds to a variety of other amyloid forming proteins. The C-terminal CPP-like PrP_23–28_ motif in its sequence, the KKRPKP [36], accompanied with the hydrophobic signal sequence may be responsible for the CPP-like property of this domain [37]. It was discussed that the chimeric signal peptide-polycationic motif hybrid peptide binds the oligomeric amyloid aggregates, and the motif could be used to target other amyloid processes. When replacing the residues 1–22 in the mouse mPrP_1–28_ with the Neural adhesion molecule-1 (NCAM1) derived sequence, the cell-penetrating properties and anti-prion effect remained and were even enhanced [9,38,39] when compared to the PrP_1–28_.

The Aβ is just one of the many amyloidogenic proteins that will, under certain conditions, associate into aggregates with a cross-β structure and fibrillary morphology. Many other conformational diseases or fatal prion diseases, be they infectious, non-infectious, or with high hereditary background neurodegenerative diseases, such as the Parkinson´s disease, the Huntington´s disease, and amyotrophic lateral sclerosis, share the AD characteristic of misfolded protein aggregation. The ability of targeted peptides to dissociate these aggregates or to reduce their aggregation would help to reduce amyloid-mediated decline in the patients and increase the life quality and expectancy. The Aβ can be used as a model for these diseases and further selective screening can be done for specific amyloidosis.

In this work we design and modify new cell-penetrating and aggregation reducing peptides based on protein sequences. These chimeric peptides are either fused with the PrP_23–28_ sequence, or the protein derived sequences are modified into CPPs. These new peptides are compared in the effect of reducing the aggregation of the Aβ, and for the most efficient, the CPP-properties are verified. We show that a mammalian cell compatible expression plasmid can be used for screening the anti-aggregation properties of compounds or peptides, without the need for additional immuno-labelling. The new peptides are compared to the PrP_1–28_ and the NCAM1_11–19_PrP_23–28_ peptides [37].

## 2. Results and Discussion

### 2.1. Peptide Design and Characterisation

**Figure 1:**
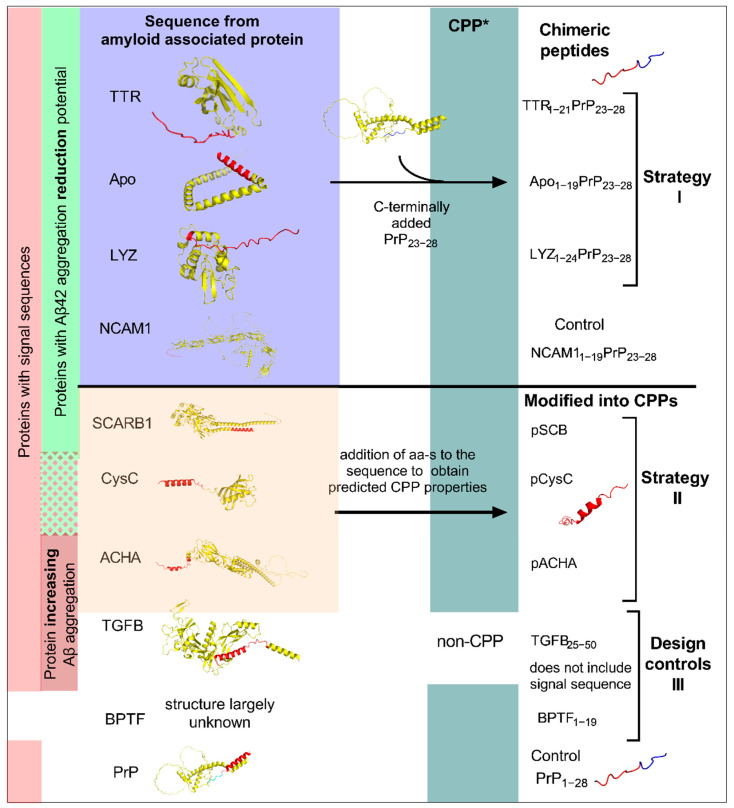
Structures of proteins used for the design of the peptides, design principle of anti-aggregation peptides, and controls. Protein identificators: mouse Transthyretin, TTR–P07309, 2QPF; mouse Apolipoprotein A-II, Apo-P09813; human Lysozyme C, LYZ–P61626; mouse Neural cell adhesion molecule 1, NCAM1– P13595; mouse major prion protein, PrP P04925; mouse Scavenger receptor class B member 1, SCARB1–Q61009; mouse Cystatin-C, CysC–P21460; mouse Neuronal acetylcholine receptor subunit alpha-7, ACHA–P49582; human Transforming growth factor beta-2 proprotein, TGFB–P61812; human Nucleosome-remodelling factor subunit BPTF, BPTF–2F6J. Firstly, proteins with signal sequences and known association with amyloids were chosen. In the case of CysC, the reduction has been associated mainly with human CysC, whereas in this work the mouse CysC is used. Depending on their suggested effected on Aβ_42_ the proteins were divided into aggregation-reducing and aggregation-increasing proteins. Secondly, predictions were made on the sequences, and specific regions and modifications were chosen to be included in the sequences. Proteins structures (yellow) have marked peptide sequences included in the peptide design (red, for specific sequence part refer to Table S2). Design was divided into two: creation of chimeric peptide by addition of the PrP_23–28_ (marked in blue) (I), or modification of the sequence into a cell-penetrating peptide (II). Additional controls besides NCAM1_1–19_PrP_23–28_ and PrP_1–28_, TGFB, and BPTF derived peptides, were used (III). These designed peptides did not contain signal sequences. *—CPP prediction was done with CPP predictor and CellPPD. The regions of the section included in the design is shown in the right column (red), chimeric peptide marked as fusion of two parts (red and blue), where the blue is the PrP_23–28_.

In this work the mouse prion-protein derived peptides PrP_1–28_ and NCAM1_1–19_PrP_23–28_ previously published by Gräslund et al. [37,38,39] serve as peptide-based controls with shown anti-prion properties (Figure 1 and Table 1). For peptide design, two strategies were introduced: (I) the addition of the PrP_23–28_ to a signal sequence of the protein with reduction effect on the Aβ, creating a chimeric peptide, and (II) the modification of (signal) sequence from the protein to obtain a predicted CPP function (Figure 1, Tables S1 and S2). For this we chose three proteins as a basis for further chimeric peptide design (Figure 1, marked with I: mouse TTR, mouse Apolipoprotein A-II, human Lysozyme C), three proteins for modified signal sequence into CPPs (Figure 1, marked with II: mouse Scavenger receptor class B member 1, mouse Neuronal acetylcholine receptor subunit alpha-7, mouse Cystatin C), and two control peptides derived from two protein sequences (Figure 1, marked III: human Transforming growth factor beta-2 pro-protein and human Nucleosome-remodelling factor subunit BPTF).

Firstly, the prediction of signal sequences from the protein was performed using Signal-IP and PrediSi servers (Table S3 and Figure S1). The proteins from which the control peptides PrP_23–28_ and NCAM1_1–19_PrP_23–28_ were derived, the PrP and the NCAM1 both included signal sequences (Table S3 and Figure S2). Secondly, the cell penetrating peptide predictor and the CellPPD were used to predict the probability of the signal sequence to be a CPP (Figure 1, Table S3). The sequences from the N-terminus of the protein did not contain predicted CPP-like sequences; therefore, further modifications were required. Additionally, the associated effect of the protein on the Aβ, the inclusions of signal sequence, and the predicted CPP properties after deriving the peptide (Table S4) were considered, including other properties listed in Table S5 and predicted secondary structures (Table S6) of the peptides. All peptides were predicted as probable anti-inflammatory peptides (Table S4).

Strategy I (Figure 1): to the sequences derived from proteins TTR, Apo, and LYZ, the PrP_23–28_ was added to the C-terminus of the peptide. For design, the most probable cleavage site (CS) was predicted from the protein sequences (Table S3 and Figure S1). For the design of chimeric TTR peptide, the residues 1–21, which also included the signal sequence and CS, from TTR were attached to the PrP_23–28_ to obtain TTR_1–21_PrP_23–28_. For ApoAII the chimeric peptide sequence was constructed by addition of residues 1-19 from the ApoAII sequence to the PrP_23-28_ sequence creating the Apo_1–19_PrP_23–28_ peptide. For LYZ the CS was predicted between the residues 18/19 (Table S3 and Figure S1), but for chimeric peptide LYZ_1–24_PrP_23–28_ an additional five residues from the protein sequence were included, with the aim of reduced solubility profile (Table S5, Figure S3), and reduction in predicted secondary structure similarity to the TTR_1–21_PrP_23–28_, and the Apo_1–19_PrP_23–28_ (Table S6). In the TTR-, Apo- and LYZ-derived chimeric peptides the predicted CPP properties were probably due to the addition of the PrP_23–28_ motif which is known to be a CPP.

Strategy II: For the CysC, SCARB1 and ACHA proteins the non-CPP signal sequences (Table S3) were modified into predicted CPPs. Although the scores were lower than for aforementioned three chimeric peptides (Table S4), all modified peptides were predicted as CPPs. The peptides designed in strategy II included additional amino acids in order to make them into the predicted CPPs. Therefore, in this work we refer to these as peptides derived from protein indicated by the “p” in front of the protein name from which it was derived.

The peptides TGFB_25–50_ and BPTF_1–19_ served as additional controls. TGFB_25–50_ was predicted as non-CPP, and BPTF_1–19_ was predicted to have CPP properties. Neither TGFB_25–50_ nor BPTF_1–19_ included a predicted signal sequence. Additional predictions for allergenicity, anticancer properties and haemolytic properties did not reveal new highlighted peptides (Table S7). Nevertheless, in the prediction of BBB penetration, the TTR_1–21_PrP_23–28_, and pCysC were predicted as probably not BBB penetrating (Table S7), whereas all other were predicted as BBB permeable. This should be considered, as the target amyloid aggregates are located in the brain; therefore, therapeutics capable of crossing the BBB would have an advantage.

The secondary structure of the peptide may help with the interactions with the Aβ, and the folding ability of the CPPs to form α-helices and β-sheets may increase their efficiency to penetrate the cells [40]. We predicted the secondary structures of the peptides (Table 1 and Table S6), and as the next step, the CD spectra of the peptides was detected and the secondary structures of the peptides in water (Figure S4b) and buffer (Figure S4c) were calculated. The peptides adapted mostly β-strand structures in water, with the highest α-helical structure percentage for the BPTF-derived peptide and the PrP_1–28_. In buffers the physiologic conditions near membrane and extracellular environment are mimicked more closely. Interestingly, the structures of the peptides were enhanced toward an increase in the helical structure, and the peptide pCysC adopted mainly the α-helical structure. The BPTF-derived peptide remained mostly in a random structure, as predicted. The pSCB peptide secondary structure was also only mildly affected (Figure S4b,c).

### 2.2. Interaction with Aβ and Reduction in Amyloid Beta Aggregation In Vitro

**Figure 2:**
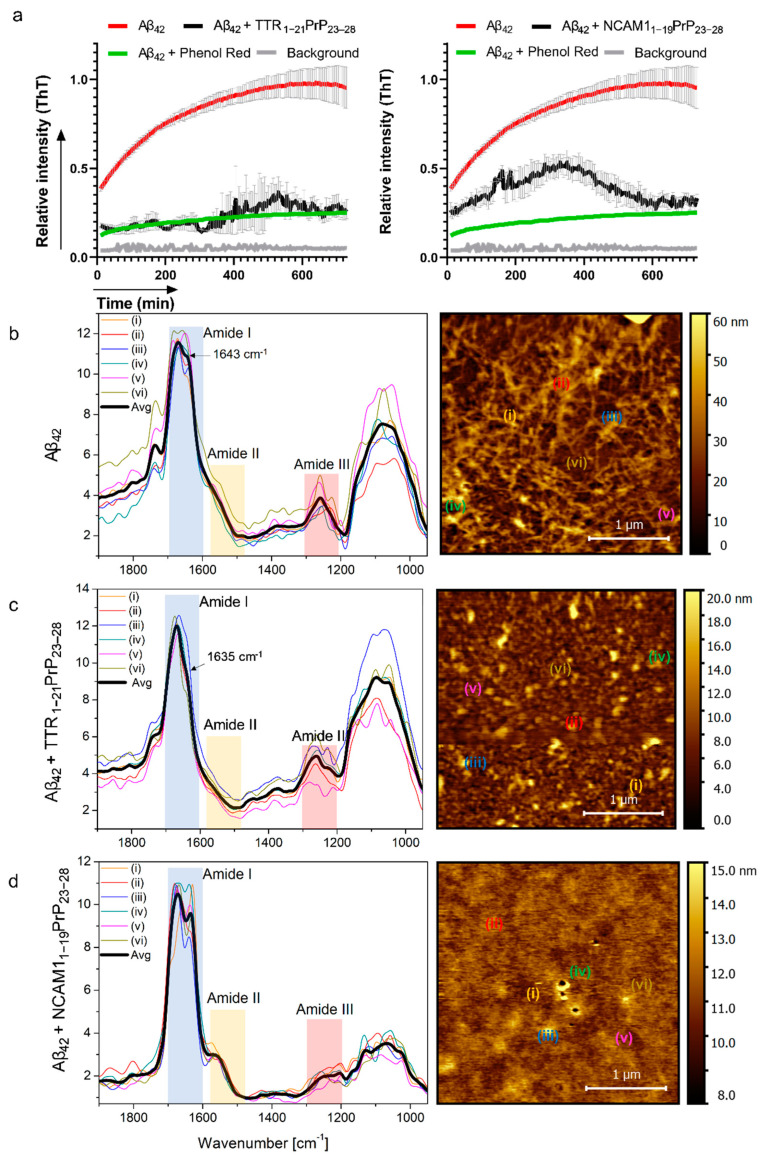
Assessment of the reduction in the aggregation of the Aβ_42_. (**a**) For detection of Aβ_42_ aggregates Thioflavin T-based assay was used. 1:1 ratio of Aβ_42_ to the peptide was used for these experiments. Results are normalized to the max intensity in the free Aβ_42_ group. Inhibition control phenol red was added at a 100 µM final concentration. Background control samples included ThT in water, ThT in buffer, and water or buffer without ThT. (**b**) AFM-infrared data from Aβ_42_ aggregation in (Aβ_42_) the absence, and in the (**c**) presence of (Aβ_42_ + TTR_1–21_PrP_23–28_) TTR_1–21_PrP_23-28_ and (**d**) (Aβ_42_ + NCAM1_1–19_PrP_23–28_ ) NCAM1_1–19_PrP_23–28_ amyloid inhibitors. The infrared spectra data have been collected from the area underneath the points numbered in the corresponding height images.

The amyloid plaques formed by the Aβ peptides consist mainly of the Aβ_40_ and the Aβ_42_. The Aβ_42_ is highly prone to aggregate, and its oligomers are described as the main driving force behind the cellular toxicity. Reduction in the aggregation of amyloid peptide in cell free, controlled conditions in vitro is often used as the first screening method. This is often the primary indication of the Aβ aggregation reduction ability of the compound. Thioflavin T- and S-based assays are used for the in vivo and the in vitro detection of amyloid aggregates, as the dye binds to the beta-sheet oligomers/aggregates, and by doing so, the ThT fluorescence intensity shifts and can be quantified. Firstly, the potential of the peptides to reduce the aggregation of the Aβ_42_ (Figure 2a, sequence in Table S8) was assessed. All the peptides were able to reduce the aggregation, except for the control peptides TGFB_25–50_ and BPTF_1–19_ (Figure S4d). The TTR-based peptide had the strongest reduction potential over the incubation time, while pACHA treated group had a slight increase in fluorescence during the incubation. The control peptide NCAM1_1–19_PrP_23–28_ also led to the reduction in the aggregation (Figure 2) at a similar 1:1 ratio of Aβ:NCAM1_1–19_PrP_23–28_ reported in previous work [39]. The designed peptides themselves, without the presence of Aβ_42_, did not lead to a significant increase in ThT signal (Figure S4d and comparison of peptides in Figure 2 can be found in Figure S4e).

The interaction of the Aβ_42_ peptide with the aggregation inhibitor peptides was further investigated using atomic force microscopy combined with infrared spectroscopy (AFM-IR). This technique has the advantage of simultaneously providing morphology visualization of the amyloid aggregates and spectroscopic information at the nanoscale, being successfully used in the characterization of the fibrillization pathway of amyloidogenic nanoparticles. Topography data from specimens containing only the Aβ_42_ peptide revealed that these samples are populated by an intricate network of fibers, consistent with the formation of amyloid plaques (Figure 2b). High resolution images from Aβ_42_ aggregates unveil that the fibers are composed of intertwined fibrils (Figure S5d). Fibers in these networks have diameters around 100–150 nm, with the lengths near to the micrometer range (Figure S5d). Infrared spectra from Aβ_42_ fibers exhibit sharp resonances across the amide I and amide III bands and can be used to devise information on the secondary structure of aggregates. The amide I band is featured by a strong resonance centered at ~1670 cm^−1^ along with a shoulder at 1643 cm^−1^, indicating the presence of β-turns and anti-parallel β-sheets [41]. A vibration centered at 1259 cm^−1^ in the amide III range is harder to interpret and is often assigned to a complex combination of COO-, C-N, C-C stretching, C-H, and N-H bending [41,42]. In the presence of the TTR_1–21_PrP_23–28_ peptide, the morphology of the aggregates dramatically changes (Figure 2c), and fibrils are no longer observed in the samples. Topography images obtained at high resolution show the complete inhibition of fibers even at the nanoscopic scale. Instead, the aggregates in the mixtures have irregular shapes and are characterized by globular geometry with sizes reaching a few hundred nanometers (Figure S5b). Like Aβ_42_ fibers, the infrared spectra display distinctive resonances across the amide range. However, a noteworthy deviation is observed in the characteristic peak at 1643 cm^−1^, which has shifted to 1635 cm^−1^. This shift indicates a reorganization of the peptide arrangement, suggesting that the presence of the TTR inhibitor has likely caused a decrease in the β-sheet content. In mixtures containing the control NCAM1_1–21_PrP_23–28_ peptide, the formation of fibers is also suppressed, as indicated by the AFM topography data shown in Figure 2d. In this case, the specimens are characterized by an anamorphous matrix encrusted with aggregates with diameters of about 300 nm. A downshift of the β-sheet peak to 1633 cm^−1^ corroborates the decrease in the fraction of these secondary conformations in the self-assemblies.

In addition, small angle X-ray scattering (SAXS) and dynamic light scattering (DLS) assays were performed to validate the AFM experiments with the advantage of analyzing the samples in solution. We found that scattering curves from mixtures containing the TTR_1–21_PrP_23–28_ peptide or the NCAM1_1–21_PrP_23–28_ peptide have similar profiles indicating that both solutions contain smaller aggregates. In contrast, SAXS data from Aβ_42_ solutions are characterized by a different profile consistent with the presence of long fibers (Figure S5) [43]. Size distributions revealed by DLS assays (Figure S6) indicate populations with hydrodynamic diameter around 815 nm in Aβ_42_ samples with a large distribution that correlates with polydisperse fibers, whereas in the presence of NCAM1_1–21_PrP_23–28_ or TTR_1–21_PrP_23–28_ the hydrodynamic diameters reduce to mainly about 280 nm, consistent with the AFM data. In addition, in agreement with AFM images, DLS data particles with sizes in the range of a few tens of nanometers when amyloid inhibitors are in the formulation. Putting the above findings together, the morphological and spectroscopic data obtained through AFM-IR, along with SAXS and DLS information obtained in liquid medium, provide direct evidence supporting the amyloid-suppressive effect exerted by these peptides, in agreement with indirect observations found in ThT fluorescence assays.

### 2.3. Reduction in Amyloid Aggregates in Cell Culture

The cells themselves and cell media, e.g., physiologic conditions may also play a role in the aggregation tendencies. The next step was to investigate if the peptides were able to reduce the aggregation of the Aβ_42_ on the cells. For this, the cells were infected with the Aβ_42_ solution mixed with aggregate seeds, followed by the addition of the peptides and co-incubation on the cells. After fixing and labelling with antibodies a bright signal was observed on the infected cells and the cells treated with pACHA, but in the other treatment groups it was hard to distinguish between the different fluorescence levels. Additionally, during immunohistochemically (IHC) fixing and staining procedure some of the aggregates were washed away; therefore, only the aggregates associated with the cells were maintained, which may have altered the possible interpretation of results (Figure S7).

Due to these shortcomings, including several steps of washes, the need of fixing the cells, and dependency on the efficacy of antibody binding required for IHC finding a suitable method that could be used for efficient screening of peptides and compounds was required. Although there are several plasmids expressing Aβ_42_ available for the expression in the yeast and the bacteria [44] the aim was to find a suitable method for the mammalian cell cultures. In one work, a fusion protein of two reporters, enhanced yellow fluorescent protein, and renilla luciferase were linked together by Aβ_40_ enabling to monitor the location and the aggregation state of the Aβ_42_ [44]. In the previous works by other groups, in the case of HEK293 cells the expressed Ub–Aβ_42_–GFP the Aβ_42_–GFP was found to accumulate in the cytosol and inside of the nuclei of the cells [45]. In another work it was shown that the mammalian expressed APP signal sequence containing the Aβ_42_ forms cytosolic aggregates after its export from the endoplasmic reticulum [46]. It has been also shown that the linker between the Aβ_42_ and the GFP fusion protein was of importance, as using shorter linkers would lead to the quenching of the fluorescent signal due to the Aβ_42_ aggregation [47].

These notions led us to believe that based on this we could compose a detection method that would simplify the screening of anti-aggregation compounds, and would be independent on the formation of the aggregates outside the cells. Based on the aim of our work, we constructed the Aβ-EGFP fusion protein and the Aβ mammalian expressing plasmids. The linker between the Aβ and the EGFP was minimized to enhance the quenching effect caused most probably by the aggregation of the product. The idea was that if the construct aggregates after expression, the fluorescent signal reduces or is quenched because of the aggregation and the improper folding of the EGFP. If the tested compound would help to reduce the aggregation, the EGFP is properly folded/unquenched, and the fluorescent signal is enhanced. Therefore, instead of loss of signal as detected with IHC, the appearance or the enhancement of the fluorescent signal is detected. Firstly, it was confirmed whether any fluorescence signal after transfecting U87 cells with Aβ-EGFP expressing plasmid was observed. As only a weak fluorescent signal was observed, it had to be confirmed that the transfection reagent is suitable for the given application and for the used conditions. For this, a small set of transfection reagents were screened based on their efficacy to deliver plasmid into the cells and lead to reporter expression (Figure S8). The highest transfected cell population was detected for NickFect51; therefore, for transfection it was chosen for further experiments. As indicated by previous works [47], it had to be verified that the lack of fluorescence signal was due to aggregation, and not because the expression vector does not express the construct. For this we used IHC labelling post-transfection to detect the expressed Aβ_42_. In both the Aβ_1–42_-EGFP and the Aβ_1–42_ expressing plasmid treated groups a signal from the fluorescently labelled anti-Aβ_42_ antibodies (Figure S9) was detected, indicating that the construct was able to express the peptide and fusion-peptide in the mammalian cells. Additionally, the fluorescence signal background for just transfected cells was low, indicating its suitability as a screening method.

### 2.4. Reduction in Expressed Aβ Aggregation in Cells

**Figure 3:**
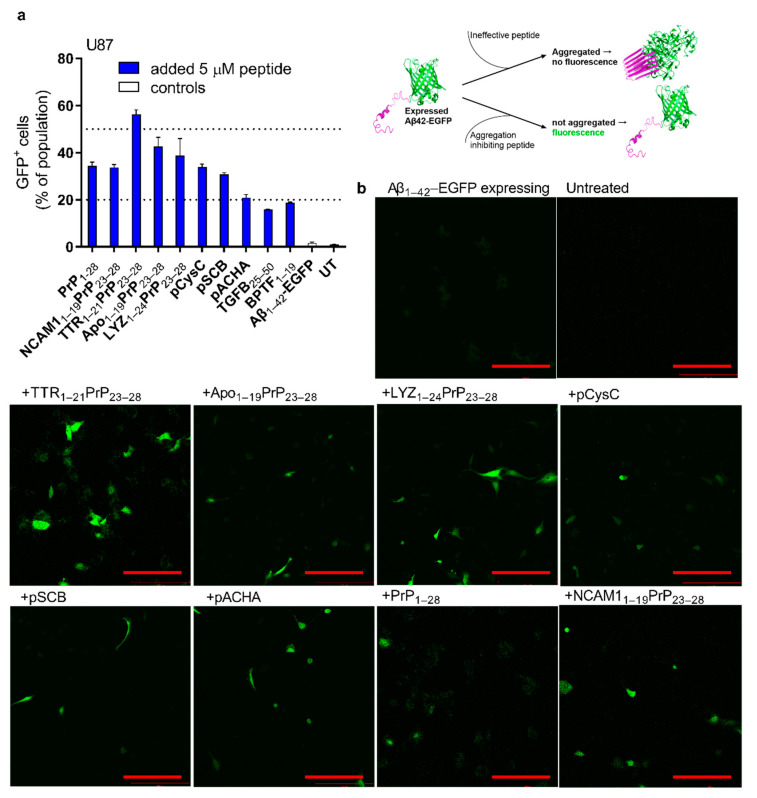
Addition of the peptides leads to the reduction in aggregation. Peptide efficacy was assessed in U87 cells transfected with Aβ_1–42_-GFP fusion protein expressing plasmid. (**a**) The reduction in the aggregation of the expressed Aβ_1–42_-GFP assessed with flow cytometry. Cells were transfected with 0.5 µg Aβ_42_–EGFP fusion protein expressing plasmid. After 2 h incubation, the cells were washed and the peptides were added at the final concentration of 5 µM to the cells. 24 h post-addition of the peptides, the cells were detached and cell population with fluorescent signal was detected. To set the threshold for GFP+ cell population, untreated cells were used. (**b**) Confocal microscopy live cell images of controls and cells treated with peptides. After transfection media was replaced with fresh media and peptides were added at the final concentration of 10 µM. 24 h post-addition of the peptides images were taken to detect fluorescent signal from the expressed and non-aggregated Aβ_42_–EGFP. Red bar 200 µm. Images were taken with 63× oil immersion objective.

The appearance of a fluorescent signal due to the reduction in aggregation was confirmed by adding known Aβ inhibitors phenol red and Morin to the Aβ_42_–EGFP expressing cells. As expected, the addition of phenol red significantly increased the green signal in the transfected cells (Figure S10a). Next, the peptides were screened with flow cytometry (Figure 3a and Figure S10b,c) and further tested regarding whether the signal was visible in confocal microscopy (Figure 3b and Figure S11). The fluorescent cell population was compared between treatment groups after the addition of 5 µM peptides and the fluorescence of the cells by confocal microscopy after expression of the Aβ_42_–EGFP fusion protein, and addition of 10 µM peptide (Figure 3). In both experiments the same peptides were highlighted. Screening in another cell line, SH-Sy5y, was also preformed, but due to the low expression efficacy and the morphological differences between the cells on the same SH–Sy5y plates, the focus laid on U87 for most experiments at this point. The Aβ_40_ peptide is less prone to aggregate; therefore, it was investigated whether this peptide could also be used for screening, and whether the results differed between the two amyloid peptides. Interestingly, in the case of Aβ_40_-EGFP expression, the LYZ_1–21_PrP_23–28_ had the highest fluorescent positive cell population, already at 5 µM peptide concentration, but the effect was not enhanced even at the higher peptide concentrations. The TTR-derived peptide and the LYZ-derived peptides had the highest percentage of fluorescence-positive cells, compared to other tested peptides. (Figure S10d).

As TTR-derived peptide seemed the most efficient in both confocal and flow cytometry-based assays, and for both Aβ-forms, it was determined whether a lower concentration would also lead to the reduction effect. For this, accompanied by confocal microscopy-based detection, the TTR-derived peptide was tested at 5 µM and 10 µM concentrations and compared to the 10 µM NCAM1_1–19_PrP_23–28_ and PrP_1–28_ peptides. (Figure S11). In both the 5 and 10 µM peptide treated groups cells gained in fluorescent signal, although the signal strength varied (Figure S11), the same was seen in the flow cytometry experiment results, where at 5 µM and 10 µM concentrations GFP+ cell population was similar (Figure S10b). This indicates that maybe not only the cell population affected should be considered, but also the total fluorescence gained after the addition of the peptides.

### 2.5. Reduction in Amyloid Toxicity and the Safety of Peptides to the Cells

The viability of the cells is reduced due to intraneuronal presence of the Aβ_42_; therefore, the reduction in toxicity of amyloid to the cells would be beneficial for cell survival and a possible reduction in clinical signs in patients. Although the experiments are performed on cultured cells, the effect of the peptides to cell viability and the possibility of reducing effects caused by Aβ_42_ was investigated.

**Figure 4:**
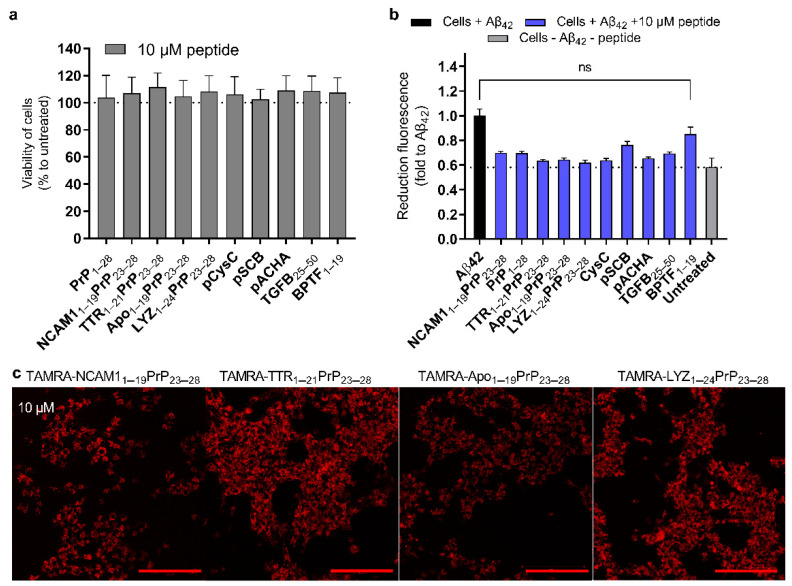
Peptides are not toxic to cells and can reduce Aβ_42_ toxicity to the cells. (**a**) Cell viability after addition of the peptides was assessed using MTS assay 24 h post-addition of peptides on U87 cells in serum containing media. The final concentration of the peptides on cells was 10 µM. Peptides were assessed in the absence of amyloid peptide. (**b**) Reduction in amyloid toxicity on U87 cells after addition of peptides, using ToxGreen assay. Results are shown as fold change to fluorescence detected from cells treated with Aβ_42._ Unpaired *t*-test with Welch correction. ns = *p* > 0.05, all other groups when compared to the Aβ_42_ group were revealed as statistically significant (not marked). (**c**) Internalization of labelled peptides 2 h post-addition to U87 cells. Confocal images were captured with 20× objective, red bar 200 µm.

Firstly, to confirm the safety of the peptides themselves, we show that the peptides did not affect the viability of the cells in serum containing media (Figure 4a). Similar results were seen in the serum containing media with other peptide concentrations (Figure S12a). Interestingly, in serum free conditions the PrP_1–28_ led to a decrease in the cell viability already at the low concentrations (Figure S12b), whereas the TTR-derived, the Apo-derived, the pCysC, and the NCAM1-derived peptides reduced cell viability only at 20 µM concentrations in the serum free media. Another toxicity assay was included in the screening, as it has been indicated in several works that the use of MTS- or MTT-based assays in combination with the Aβ may lead to misinterpretation of the results. The aim was to find any indications of the changes in the viability of the cells after the addition of the peptides. The viability of cells was not reduced due to the addition of inhibition peptides (Figure S13a). In the case of the PrP_1–28_, the reduction in the viability of cells in serum free media was observed at already at 2.5 µM peptide concentration, and over 20% reduction in the case of the 20 µM peptide (Figure S12b).

In addition to the effect of the peptides on the cell viability, we investigated whether the Aβ_42_ toxicity on the cells could be reduced by adding our inhibition peptides. It was observed that there was a reduction in toxicity with most peptides, except for the BPTF_1–19_ (Figure 4b and Figure S13b). Interestingly, the pACHA, which was highlighted in ThT assay (Figure 2), seemed inefficient in IHC assay, and inefficient in mammalian cell-based assay, was able to reduce the Aβ_42_ toxicity on the cells (Figure 4b). This may be due to the pACHA ability to interact with the Aβ_42,_ but because of to its poor CPP properties it is not efficient in the other cell experiments. As in the toxicity reduction experiment, the Aβ was added into the media, the pACHA effect could be more apparent, although due to IHC experiment results (Figure S7) the mechanism requires further investigation.

### 2.6. The Chimeric Peptides Efficient in Mammalian Cell Test System Have CPP Properties

Based on the predicted CPP scores (Table S4) and results with reduction in aggregation fluorescently labelled NCAM1_1–19_PrP_23–28,_ TTR_1–21_PrP_23–28_, Apo_1–19_PrP_23–28_, LYZ_1–24_PrP_23–28_ peptides were synthesized and their internalization into the cells was tested. The PrP_23–28_ sequence is a CPP; therefore, the addition of this sequence to the signal sequences should make the chimeric peptides also CPPs. In addition to these predicted CPPs, the BPTF_1–19_ was synthesized and its cellular internalization tested. Compared to the chimeric peptides, the BPTF_1–19_ was not internalized as efficiently as the NCAM1_1–19_PrP_23–28_, the TTR_1–21_PrP_23–28_, the Apo_1–19_PrP_23–28_, and the LYZ_1–24_PrP_23–28_ as indicated by the fluorescence intensities (Figure 4c and Figure S14). Nevertheless, at higher concentration and also longer time-point the BPTF derived peptide was also internalized and has confirmed CPP properties (Figure S15).

## 3. Materials and Methods

### 3.1. Vizualisation and Analysis

Graph compilation, analysis, and statistical analysis was performed using GraphPad Prism version 9.5.1 for Windows, GraphPad Software, San Diego, CA, USA, www.graphpad.com (accessed on 18 February 2023). For protein visualization the PyMOL Molecular Graphics System, Version 2.0 Schrödinger, LLC (New York, NY, USA) was used.

### 3.2. Predictions and Calculations

For predictions and calculations the following tools and servers were used. Prediction of signal sequences was performed with SignalIP 5.0 (https://services.healthtech.dtu.dk/service.php?SignalP-5.0, accessed on 1 September 2021) [48], and PrediSi (http://www.predisi.de/, accessed on 1 September 2021). For prediction of CPP probability/property of the peptide, the cell-penetrating peptide predictor [49], CellPPD (http://crdd.osdd.net/raghava/cellppd/, accessed on 1 September 2021) [50], and MLCPP 2.0 (https://balalab-skku.org/mlcpp2/ (accessed on 22 March 2022) [51], were used. The solubility of the peptides was predicted using CamSol v2.2 (http://www-vendruscolo.ch.cam.ac.uk/camsolmethod.html (accessed on 1 September 2021) [52,53] and Peptide Property Calculator ver 3.1 from Biosyn (https://www.biosyn.com/peptidepropertycalculator/peptidepropertycalculator.aspx (accessed on 1 January 2022). The net charge of the peptide was calculated with PepCalc.com. The secondary structure of the peptides was predicted with Peptide Property Calculator ver 3.1 from Biosyn, S2Dv2 (https://www-cohsoftware.ch.cam.ac.uk/index.php/s2D (accessed on 1 September 2021) [52,53], and predictor based on [54,55,56], accessible from http://cib.cf.ocha.ac.jp/bitool/MIX/ (accessed on 1 September 2021). Additional predictions were made with CSM peptide (https://biosig.lab.uq.edu.au/csm_peptides/, accessed on 5 November, 2022) [57], ToxinPred (https://webs.iiitd.edu.in/raghava/toxinpred/algo.php, accessed on 1 September 2021) [58,59], HemoPI (https://webs.iiitd.edu.in/raghava/hemopi/design.php, accessed on 1 September 2021) [60], AllerTOP v.2. (https://www.ddg-pharmfac.net/AllerTOP/, accessed on 1 September 2021) [61], and AllergenFP v.1.0 (http://ddg-pharmfac.net/AllergenFP/, accessed on 1 August 2022). The BBB crossing potential of the peptide was predicted using BBPpredict (http://i.uestc.edu.cn/BBPpredict/cgi-bin/BBPpredict.pl, accessed on 1 August 2022) [62].

### 3.3. Peptide Synthesis

Peptides were synthesized on an automated peptide synthesizer (Biotage Initiator+ Alstra) using the fluorenylmethyloxycarbonyl (Fmoc) solid phase peptide synthesis strategy with Rink-amide ChemMatrix resin (0.4 mmol g^−1^ loading) to obtain C-terminally amidated peptides. For PrP_23–28_ containing peptides the resin was downloaded to 0.2 mmol/g while coupling the first amino acid. For the fluorescently labelled peptides, 5(6)-Carboxyfluorescein (FAM) or 5(6)-Carboxytetramethylrhodamine (TAMRA) was coupled manually to the N-terminus of the peptide overnight, at room temperature. The removal of the protective groups (Fmoc) was done with 20% piperidine in dimethylformamide (DMF). The reaction was carried out in DMF using HOBT/TBTU (1-hydroxybenzotriazole/2-(1H-benzotriazol-1-yl)-1,1,3,3-tetramethyluronium tetrafluoroborate) for manual or DIC/Oxyma (N,N′-diisopropylcarbodiimide/ethyl 2-cyano-2-(hydroxyimino)acetate) for machine synthesis as coupling reagents, with DIEA (diisopropylethylamine) as an activator base. Cleavage was performed with trifluoroacetic acid, 2.5% triisopropylsilane and 2.5% water for 2 h at room temperature, for peptides including Cys, Met in the sequence, 2.5% EDT (ethanedithiol) was added to the cleavage mix. Peptides were purified by reversed-phase high-performance liquid chromatography on a C3 column (ZORBAX 300SB-C3, 5 μm, 300 Å, 250 × 9.4 mm) using a gradient of acetonitrile/water containing 0.1% TFA (trifluoroacetic acid). The molecular weight of the peptides was analyzed by matrix-assisted laser desorption-ionization/time of flight mass spectrometry (Bruker Daltonics GmbH & Co. KG, Bremen, Germany). The concentration of the peptides was determined based on dilutions of accurately weighed substances and absorption of tyrosine, where applicable.

### 3.4. Secondary Structures of Peptides-Circular Dichroism (CD) Spectra

CD spectroscopy was used to determine the secondary structure of the peptides. CD spectra were recorded on a Chirascan CD spectrometer (Applied Photophysics, Leatherhead, UK). Signal was recorded for wavelength interval between 185 nm and 260 nm, using a 1 nm bandwidth. A quartz cuvette (Hellma Analytics, Müllheim, Germany) with an optical path length of 1 mm was used. The concentration of the peptides was 100 µM in ultrapure water or in sodium phosphate buffer, pH 7.0. Control spectra were recorded for samples containing the Aβ_42_ (50 µM) in MQ or in a buffer. The background spectrum of the solvent was subtracted from all spectra. Estimation of the α-helix and the β-strand content of the peptides, from their CD spectra, was calculated using the K2D2 algorithm and online tool BeStSel [63].

### 3.5. Nanoscale Characterization of Interactions and Reduction in Aβ_42_ Aggregation-AFM-IR, SAXS, and DLS

Atomic force microscopy measurements combined with infrared nanospectroscopy (AFM-IR) were performed on an Anasys NanoIR2-s microscope as described elsewhere [64]. Stock solutions containing the Aβ_42_ peptide and amyloid inhibitors were prepared in ultrapure water, and immediately after the dissolution of peptides, they were mixed at 1:1 ratio to a final Aβ_42_ concentration of 5 mg/mL. To assist dissolution, samples were mixed and submitted to ultrasonication for about 5 min. At this stage, we obtained clear solutions indicating full dissolution of peptides and, in the course of a few hours, slight turbidity appeared in the medium indicating the presence of fibers in suspension. These mixtures were left at rest overnight at room temperature to allow the completion of the aggregation pathway. Droplets from these solutions were cast on Au-coated silica substrates, left at rest for about 5 min, and the excess of water was removed with filter paper. The substrates with the samples were incubated within a desiccator for 24 h to ensure complete dehydration prior to further analysis. Topography images of the surfaces were collected in contact mode across areas of 3 μm^2^ × 3 μm^2^ at a resolution of 256 × 256 pixels using ContGB-G probes (spring constant 0.2 N/m, tip radius 25 nm). Infrared spectra from nanoscopic aggregates were registered by positioning the cantilever tip on the top of the particle of interest, and the samples were illuminated by infrared lasers covering wavenumbers situated between 950 and 1920 cm^−1^ (resolution of 2 cm^−1^). In this set up, the spectroscopic data contained vibrational information from a circular area with a diameter of 50 nm underneath the tip. All data were baseline subtracted (Au profile) and smoothed using an FFT filter (7 points of window). Image enhancement and profile measurements were carried out using the software Gwyddion. Further high-resolution images were obtained on a Bruker Multimode 8-HR microscope operating in PeakForce tapping mode, which is especially suited for topography analyzes of soft materials [65]. The cantilever was equipped with ultrathin Scanasist-air probes (spring constant of 0.4 N/m, tip radius 2 nm).

Small-angle X-ray scattering (SAXS) were done in a Bruker Nanostar equipment. The samples were prepared as above; however, measurements have been started about 3 h after mixing. The samples were placed in 1.5 mm quartz capillaries distant from detector in 661 mm and irradiated 6 times with an X ray beam with 𝜆 = 1.54 Å for 1800 s at room temperature. The scattering intensity was normalized to exclude the scattering from capillaries and water. Data were analyzed using SUPERSAXS package (C. L. P. Oliveira and J. S. Pedersen, unpublished).

Dynamic light scattering (DLS) analyses were performed in a Brookhaven Instruments Corporation 90Plus Particle Size Analyzer. The samples used on SAXS experiments were diluted to a final concentration of Aβ_42_ of 0.85 mM. The experiments were carried out at 25 °C. For each sample five runs of 2 min were performed. The correlation curve obtained was analyzed using the CONTIN method on the 9KDLSW BIC Particle Sizing Software.

### 3.6. Reducing Aggregation of Aβ_42_-Thioflavin T Assay

For detection of the Aβ aggregation, SensoLyte^®^ Thioflavin T ß-Amyloid (1-42) Aggregation Kit was used. For this Thioflavin T was mixed with the assay buffer according to the manufacturer’s instructions. The Aβ solution was prepared by adding ice-cold buffer to the lyophilized peptide powder, then sonicated for 5 min and then centrifuged at 10,000 rpm, for 5 min at 4 °C. The supernatant was used for the assay. The aggregation inhibiting control solutions at 20 mM concentration Phenol Red and Morin solution were used as controls. To each well 10 µL of 2 mM ThT was added, 5 µL of test samples (peptides, inhibitors or MQ), and 85 µL of Aβ_42_ solution in buffer. For each peptide, a set of controls in the ThT solution without Aβ_42_ was included. Instead of Aβ_42_ 85 µL buffer was added to the samples. The final volume of each well was 100 µL. Measurement was started after 10 min incubation at 37 °C with *λ*_ex_ = 440 nm, *λ*_em_ = 484 nm and shaking prior each measurement. The readings were done with 5 min intervals (starting from the start or reading) with fluorimeter (SynergyMx, BioTek, Winooski, VT, USA).

### 3.7. Cell Culture Maintenance

The U87 human glioma cells were grown on a gelatinized (0.1% solution made from gelatin from porcine skin powder (Sigma, G1890), autoclaved) dish at 37 °C and 5% CO_2_ in Dulbecco’s Modified Eagle’s Medium (DMEM) (Sigma, Darmstadt, Germany). Human neuroblastoma SH-Sy5y cells were grown in the same media, but on plates without gelatin. In both cases the media was supplemented with 0.1 mM non-essential amino acids, 1.0 mM sodium pyruvate, 100 U mL^−1^ penicillin and 100 mg mL^−1^ streptomycin, and with 10% fetal bovine serum (FBS).

### 3.8. Plasmids

The green fluorescent protein expressing plasmid pEGFP-C1 (Clontech) was used to test the transfection efficacy of transfection reagents. For the other experiments the pcDNA3.1(+) mammalian expression vector (Invitrogen, Waltham, MA, USA) with inserts were used. The inserts encoding Aβ_1–42_, mAβ_1–40_ or Aβ_1–42_, Aβ_1–40_ fused with GFP were ordered from GeneUniversal. The inserts encoding Aβ_1–42_, Aβ_1–40_ or Aβ_1–42_, Aβ_1–40_ fused with GFP were inserted into the pcDNA3.1 plasmid backbone using BamHI and NotI restriction sites under the control of strong CMV promotor (exemplified in Figure S16).

### 3.9. Complex Formation for Transfection and Transfection

The commercial transfection reagents Metafectene (Biontex, München, Germany) and Lipofectamine 3000 (Thermo Fisher Scientific, Vantaa, Finland) were used according to the manufacturer’s recommendations. The CPP NickFect51 (NF51, sequence shown in Table S8) was used at CPP/plasmid charge ratio 2:1 or 3:1 as suggested in [66], considering the positive charges from the peptide and negative charges from the plasmid backbone. The plasmid and peptide were mixed in ultrapure water, incubated at room temperature for 30 min and then added to the cells in fresh serum containing or serum free media. At 2–4 h post-transfection the media was replaced with fresh serum containing media and further incubated. Flow cytometry was used to detect fluorescent positive cell population.

### 3.10. Detection of Aβ_42_ Aggregates on Cells-IHC

Nunc™ Lab-Tek™ (Thermo Scientific™, Vantaa, Finland) chamber plates were used for seeding 30,000 U87 cells in 250 µL of media per well, one-two days prior to the experiment. On experiment day the media was replaced with fresh media. For experiments with the Aβ peptide (Sequences shown in Table S8), the peptide was added to the cells, following the addition of the other peptides at shown final concentrations. After incubation the fixing of the cells and the labelling of the Aβ were performed. For the experiments with the aim of showing the expression from the plasmid, the cells were transfected with the Aβ_42_ or the Aβ_42_-EGFP expressing plasmid, incubated, then the media was changed and the cells in fresh media were incubated further. After incubation the fixing and labelling were performed.

For IHC labelling the cells were washed with 1 × PBS and fixed with 4% paraformaldehyde in PBS, pH 7.4, for 10 min at room temperature. Following three consecutive washes with ice-cold 1 × PBS. For permeabilization of the cell membrane, the samples were incubated for 10 min with 1 × PBS containing 0.2% Triton X100. Followed by three consecutive washes for 5 min with 1 × PBS. For blocking 10% Normal Goat Serum (Abcam, ab7481) was used. For this the samples were incubated with the serum solution for 30 min. Primary antibody Anti-Amyloid beta 1-42 antibody (Abcam, ab10148), was diluted in 1 × PBS containing 1% bovine serum albumin (BSA), and 0.2% Triton X100 overnight in dark at 4 °C. The solution was then decanted, and the samples were washed three times for 5 min with 1 × PBS. The secondary antibody AlexaFlour 488 goat anti-rabbit (Thermo, A11008) or Alexa Flour 568 goat anti-rabbit IgG H + L was diluted in 1 × PBS containing 1% BSA and 0.2% Triton X100. Then incubated with the samples for 1 h in the dark. The secondary antibody solution was then decanted, followed by washes with 1 × PBS for three times for 5 min.

Where applicable, for counterstaining the cells were incubated with 0.3 μg/mL DAPI for 1 min, followed by several washes. After washes, the 1 x PBS was added to the sample wells. Samples were visualized with Zeiss LSM710 (Carl Zeiss AG, Oberkochen, Germany). For detecting the fluorescent label from Aβ_42_ a 488 nm laser, and 493-490 filter were used, and for detecting the nuclei a 405 nm laser with the 416-503 filter was used. Images were taken with the 20× magnification or the 62× oil immersion magnification.

### 3.11. The Internalization of the Labelled Peptides and the Reduction in Aβ_42_ Aggregation Visualized on Live Cells Using Confocal Microscopy

Nunc™ Lab-Tek™ (Thermo Scientific™, Vantaa, Finland) chamber plates were used to seed cells one day prior to the experiment. The 3 × 10^4^ cells were seeded in a serum containing media and incubated for 1–2 days to allow attachment of the cells. For internalization experiments, the media was changed on the cells and fluorescently labelled peptides were added to the cells and further incubated for 2–4 h followed by live cell imaging.

For aggregation reduction experiments the cells were transfected with NF51/pDNA complexes formed at charge ratio 2.5:1 with peptide in excess and in 1/10th of final volume on the cells. Plasmid expressing the Aβ_1–42_ fused with EGFP was used. Transfection was done in serum free media. At 2 h post-transfection the media was replaced with fresh serum containing media, and depending on the experiment, the peptides were added at given concentrations and further incubated.

Live cells were visualized with Zeiss LSM710. For detecting the fluorescence from the expressed and not-aggregated Aβ-GFP a 488 nm laser, and 493-490 filter were used, and where required, for detecting the nuclei a 405 nm laser with 416-503 filter were used. Images were taken with the 20× or the 62× magnification.

### 3.12. Reduction in the Aβ_42_ Aggregation Assessed with Flow Cytometry

For flow cytometry 5–7.5 × 10^4^ U87 or SH-Sy5y cells were seeded 24–48 h prior experiment on a 24-well plate. On experiment day, the media was replaced with fresh media and the cells were transfected with 0.5 µg of EGFP encoding plasmid pEGFP-C1, or Aβ_1–42_-GFP, Aβ_1–40_-GFP encoding plasmid. After 2 h, the media was replaced with serum containing media and the peptides were added at a 0, 2.5, 5, 7.5 or 10 µM final concentration and the cells were further incubated in dark, at 37 °C and 5% CO_2_. At 24 h post-addition of the peptides, cells were detached with 0.25% Trypsin-EDTA and suspended in 1% FBS containing 1 x PBS buffer pH 7.4. The population of the fluorescent cells was detected with Attune flow cytometer. The threshold was set based on the fluorescent cell population in the untreated cells (was 0–5%), depending on the plasmid and experimental conditions.

### 3.13. Cell Viability Detected with MTS

Cell proliferation was analyzed with the CellTiter 96^®^ Aqueous Non-Radioactive Cell Proliferation Assay (MTS) (Promega Biotech AB, Stockholm, Sweden) according to the manufacturer’s instructions. For this 1 × 10^5^ U87 cells were seeded one day prior to the experiment on a transparent 96-well plate. On the experiment day, the media was replaced with 90 μL of fresh serum free or serum (10% FBS) containing media and to the wells, the peptides at the concentration range of 0–20 µM were added. The absorbance of formazan product was measured 20 h post-addition of peptides at 490 nm with Tecan Sunrise microplate absorbance reader (Tecan Group Ltd., Stockholm, Sweden). The results are shown as fold change compared to the untreated cells (UT) to which only water was added instead of peptide solutions. Additional background controls: MTS–reagent and media, without cells, Media–cells with only media and without reagent.

### 3.14. Proliferation of Cells Detected with CellTox Green Cytotoxicity Assay

For determining proliferation of cells treated with Aβ_42_ the CellTox Green Cytotoxicity Assay (Promega, Stockholm, Sweden) was used. As determined by suggested screening of suitable experimental conditions, 2 × 10^4^ U87 cells were seeded on black 96-well plate 48 h prior experiment, in 100 µL of media. At 24 h post-seeding the media was changed to fresh media and further incubated for 24 h at 37 °C and 5% CO_2_. On the experiment day, the media was replaced with fresh serum containing media. Aβ_42_ peptide at the 5 µM final concentration or 5 µM Aβ_42_ peptide with 10 µM final concentration of designed peptides were added to the cells and further co-incubated for at 37 °C and 5% CO_2_. The delay between adding Aβ_42_ and designed peptides was 2 h. As controls 0.1% Triton X100 treated cells (dead cells), cells with water added (live cells) and cell free wells with media (background) were used.

At 15 min prior 24 h time-point post addition of peptides CellTox Green Reagent working solution was prepared freshly and to each well 100 µL reagent was added, and incubated at room temperature in the dark. Fluorescence was measured from each well by a fluorimeter (*λ*_ex_ = 488 nm, *λ*_em_ = 520 nm) (SynergyMx, BioTek).

## 4. Conclusions

The reduction in amyloid aggregation rate, reducing its toxicity to cells, and limiting neuro-inflammation, has emerged as one of the possible strategies to alleviate the ND progression of the AD or other aggregation diseases. In this work, new peptides with the CPP properties were designed and shown to reduce the aggregation of Aβ in vitro and on the cell culture models. Out of these peptides, the previously published NCAM1_1–19_PrP_23–28_ designed by Gräslund et al. [37,38,39], and the newly designed TTR_1–21_PrP_23–28_, Apo_1–19_PrP_23–28_, and LYZ _1–24_PrP_23–28_ shown in this work, are efficient in reducing the amyloid aggregation. The newly designed peptides could be optimized even further to achieve higher efficacy. The TTR- and LYZ- derived peptides emerged as the most promising candidates, although other predicted CPP peptides, such as the pCysC and the pSCB reduced the aggregation of the Aβ_42_. These peptides were assessed by using traditional methods such as ThT assay and labelling with antibodies, but also with integrated mammalian cell compatible expression on live cells. This assessment approach could be used for wider screening of anti-aggregation properties of the CPPs, and other membrane-permeable compounds. The peptides were predicted as anti-inflammatory peptides, and more importantly, predicted to have BBB penetration properties. These properties, in addition to the CPP and the anti-aggregation properties, may open new treatment opportunities for the AD. The effect of the peptides on the reduction in inflammation and crossing the BBB should be investigated further.

## Figures and Tables

**Table 1:** Protein-derived peptides used in this work, their predicted structures and net charge.

Peptide Name	Peptide Sequence ^1,2^	Net Charge ^3^	Mw ^4^
PrP_1–28_	MANLGYWLLA**LFVTM**WTDVGLCKKRPKP	3.9	3253
NCAM1_1–19_PrP_23–28_	MLRTKDL**I**W**T**L**F**FLGTAVSKKRPKP	6	2946
TTR_1–21_PrP_23–28_	MA**SLRLFLLCLAG**LVFVSEAGKKRPKP	4.9	2944
Apo_1–19_PrP_23–28_	MK**LLAMVALLVTICSL**EGAKKRPKP	4.9	2712
LYZ_1–24_PrP_23–28_	MK**ALIVLGLV**LLSVTVQGKVFERCKKRPKP	6.9	3352
pCysC	MAS**PLRSLLFLLAVLAVAW**AATPKQGPRKK	6	3235
pSCB	MGGSSR**ARWVALGLGALGLLFAAKKRA**	6	2759
pACHA	MCGRRGG**IWLALAAALLHVS**LQRRPK	6	2875
TGFB_25–50_	STLD**MDQFMRKRIEAIRGQILSKL**KL	4	3089
BPTF_1–19_	MRGRRGRPPKQPAAPAAER	6	2085

^1^ all peptides are C-terminally amidated, and N-terminus is unmodified. ^2^ Secondary structure elements of the peptide sequence were predicted using s2Dv2. Predicted secondary structures marked in the sequence: predicted β-strand, **predicted α-helix.** ^3^ Calculated net charge of the amidated peptide at pH 7.0, for calculation PepCalc.com was used. ^4^ Molecular weight of the peptides [g/mol].

## Data Availability

Data is available upon reasonable request from the corresponding author.

## Supplementary Materials

The following supporting information can be downloaded at: https://www.mdpi.com/article/10.3390/ijms24054277/s1, References [48,49,50,51,52,53,54,55,56,57,58,59,60,61,62,63,66] are cited in the supplementary materials.
