# Supplementary material for: Aggregation Limiting Cell-Penetrating Peptides Derived from Protein Signal Sequences
Source: Int J Mol Sci. 2023 Feb 21;24(5):4277. doi: 10.3390/ijms24054277 (PMC10002422; doi:10.3390/ijms24054277)
Supplement: Supplementary file 1 [file ijms-24-04277-s001.zip › ijms-2206726-supplementary.pdf]

Supplementary information:

Aggregation limiting cell-penetrating peptides derived from protein signal sequences

Ly Porosk [1,*], Heleri Heike Härk [1], Renata Naporano Bicev [2], Ilja Gaidutšik [1], Jekaterina Nebogatova [1], Eger-Jasper Armolik [1], Piret Arukuusk [1], Emerson R. da Silva [2] and Ülo Langel [1]

[1]University of Tartu, Institute of Technology, Nooruse 1, Tartu, Estonia ly.porosk@ut.ee

[2]Departamento de Biofísica, Universidade Federal de São Paulo, São Paulo 04023-062, Brazil

*Correspondence: ly.porosk@ut.ee

Predictors and calculators used for this work

Signal sequence prediction:

- SignalIP: https://services.healthtech.dtu.dk/service.php?SignalP-5.0 [1]
- PrediSi: http://www.predisi.de/

Prediction of cell-penetrating property:

- CellPPD: http://crdd.osdd.net/raghava/cellppd/ [2]
- CPP predictor: no link available [3]
- MLCPP 2.0 https://balalab-skku.org/mlcpp2/ [4]

Prediction of solubility:

- CamSol v2.2: http://www-vendruscolo.ch.cam.ac.uk/camsolmethod.html [5,6]
- PPC ver 3.1. https://www.biosyn.com/peptidepropertycalculator/peptidepropertycalculator.aspx

Prediction of peptide secondary structure:

- Predictor 1: PPC 3.1
- Predictor 2: http://cib.cf.ocha.ac.jp/bitool/MIX/ (predictor based on:[7,8,9])
- Predictor 3: S2Dv2: https://www-cohsoftware.ch.cam.ac.uk/index.php/s2D [5,6]

Prediction/calculation of secondary structure based on CD spectra:

- BeStSel: https://bestsel.elte.hu/index.php [10]

Additional predictions:

- Prediction of therapeutic potential: CSM peptide [11] https://biosig.lab.uq.edu.au/csm_peptides/results/166452752712
- Analysis/prediction of toxicity and allergenicity: ToxinPred [12,13] https://webs.iiitd.edu.in/raghava/toxinpred/algo.php
- Analysis/prediction of hemolytic acitivity: HemoPI: https://webs.iiitd.edu.in/raghava/hemopi/design.php [14]
- Analysis/prediction of allergenicity: AllerTOP, AllergenFP [15]: https://www.ddg-pharmfac.net/AllerTOP/ and http://ddg-pharmfac.net/AllergenFP/
- Prediction of Blood-Brain-Barrier crossing potential: BBPpredict,: http://i.uestc.edu.cn/BBPpredict/cgi-bin/BBPpredict.pl. [16]

References in supplementary:

1. Almagro Armenteros, J.J.; Tsirigos, K.D.; Sønderby, C.K.; Petersen, T.N.; Winther, O.; Brunak, S.; Von Heijne, G.; Nielsen, H. SignalP 5.0 improves signal peptide predictions using deep neural networks. *Nat. Biotechnol.* **2019**, *37*, 420–423. https://doi.org/10.1038/s41587-019-0036-z.
2. Gautam, A.; Chaudhary, K.; Kumar, R.; Sharma, A.; Kapoor, P.; Tyagi, A.; Open Source Drug Discovery Consortium; Raghava, G.P.S. In silico approaches for designing highly effective cell penetrating peptides. *J. Transl. Med.* **2013**, *11*, 74. https://doi.org/10.1186/1479-5876-11-74.
3. Hällbrink, M.; Kilk, K.; Elmquist, A.; Lundberg, P.; Lindgren, M.; Jiang, Y.; Pooga, M.; Soomets, U.; Langel, Ü. Prediction of Cell-Penetrating Peptides. *Int. J. Pept. Res. Ther.* **2005**, *11*, 249–259. https://doi.org/10.1007/s10989-005-9393-1.
4. Manavalan, B.; Patra, M.C. MLCPP 2.0: An Updated Cell-penetrating Peptides and Their Uptake Efficiency Predictor. *J. Mol. Biol.* **2022**, *434*, 167604. https://doi.org/10.1016/j.jmb.2022.167604.
5. Sormanni, P.; Aprile, F.A.; Vendruscolo, M. The camsol method of rational design of protein mutants with enhanced solubility. *J. Mol. Biol.* **2015**, *427*, 478–490. https://doi.org/10.1016/j.jmb.2014.09.026.
6. Sormanni, P.; Amery, L.; Ekizoglou, S.; Vendruscolo, M.; Popovic, B. Rapid and accurate in silico solubility screening of a monoclonal antibody library. *Sci. Rep.* **2017**, *7*, 8200. https://doi.org/10.1038/s41598-017-07800-w.
7. Peter, P., Jr.; Gerald, D.F. Chapter 9: Chou-Fasman Prediction of the Secondary Structure of Proteins: The Chou-Fasman-Prevelige Algorithm. In *Prediction of Protein Structure and the Principles of Protein Conformation*; Gerald, D.F., Ed.; Plenum: New York, NY, USA, 1989; pp. 391–416; ISBN 0306431319.
8. Garnier, J.; Osguthorpe, D.; Robson, B. Analysis of the accuracy and implications of simple methods for predicting the secondary structure of globular proteins. *J. Mol. Biol.* **1978**, *120*, 97–120. https://doi.org/10.1016/0022-2836(78)90297-8.
9. Qian, N.; Sejnowski, T.J. Predicting the secondary structure of globular proteins using neural network models. *J. Mol. Biol.* **1988**, *202*, 865–884. https://doi.org/10.1016/0022-2836(88)90564-5.
10. Micsonai, A.; Wien, F.; Kernya, L.; Lee, Y.-H.; Goto, Y.; Réfrégiers, M.; Kardos, J. Accurate secondary structure prediction and fold recognition for circular dichroism spectroscopy. *Proc. Natl. Acad. Sci. USA* **2015**, *112*, E3095–103. https://doi.org/10.1073/pnas.1500851112.
11. Rodrigues, C.H.M.; Garg, A.; Keizer, D.; Pires, D.E.V.; Ascher, D.B. CSM-peptides: A computational approach to rapid identification of therapeutic peptides. *Protein Sci.* **2022**, *31*, e4442. https://doi.org/10.1002/pro.4442.
12. Gupta, S.; Kapoor, P.; Chaudhary, K.; Gautam, A.; Kumar, R.; Raghava, G.P.S. Peptide Toxicity Prediction. **2014**, *1268*, 143–157. https://doi.org/10.1007/978-1-4939-2285-7_7.
13. Gupta, S.; Kapoor, P.; Chaudhary, K.; Gautam, A.; Kumar, R.; Raghava, G.P.S.; Open Source Drug Discovery Consortium. In Silico Approach for Predicting Toxicity of Peptides and Proteins. *PLoS ONE* **2013**, *8*, e73957. https://doi.org/10.1371/journal.pone.0073957.
14. Chaudhary, K.; Kumar, R.; Singh, S.; Tuknait, A.; Gautam, A.; Mathur, D.; Anand, P.; Varshney, G.C.; Raghava, G.P.S. A Web Server and Mobile App for Computing Hemolytic Potency of Peptides. *Sci. Rep.* **2016**, *6*, 22843. https://doi.org/10.1038/srep22843.
15. Dimitrov, I.; Bangov, I.; Flower, D.R.; Doytchinova, I. AllerTOP v.2—A server for in silico prediction of allergens. *J. Mol. Model.* **2014**, *20*, 2278. https://doi.org/10.1007/s00894-014-2278-5.
16. Chen, X.; Zhang, Q.; Li, B.; Lu, C.; Yang, S.; Long, J.; He, B.; Chen, H.; Huang, J. BBPpredict: A Web Service for Identifying Blood-Brain Barrier Penetrating Peptides. *Front. Genet.* **2022**, *13*, 845747. https://doi.org/10.3389/fgene.2022.845747.
17. Arukuusk, P.; Pärnaste, L.; Oskolkov, N.; Copolovici, D.-M.; Margus, H.; Padari, K.; Möll, K.; Maslovskaja, J.; Tegova, R.; Kivi, G.; et al. New generation of efficient peptide-based vectors, NickFects, for the delivery of nucleic acids. *Biochim. et Biophys. Acta (BBA) - Biomembr.* **2013**, *1828*, 1365–1373, https://doi.org/10.1016/j.bbamem.2013.01.011.

Supplementary methods:

Method S1: Analysis of peptides using UPLC

For analysis, ACQUITY UPLC H-class (Waters, USA) was used, accompanied with Empower software. The gradient was set from 5 – 95 % acetonitrile/water supplemented with 0.1% TFA, over a period of 13 min. C18 column was used with temperature set to 40°C.

Supplementary Tables:

Table S1. Peptide and protein structures used for PyMOL and visualization.

	Peptide/protein used in this work	Name of the protein	Code, name, identifier	Species
Aβ42 structures	Aβ42 monomer	Amyloid beta 1-42 peptide, human (monomer)	6SZF	Homo sapiens
	Aβ42 oligomer	Amyloid beta 1-42 peptide, human (beta sheet, oligomer)	2BEG	Homo sapiens
	Aβ42 fibril	Amyloid beta 1-42 peptide, human (protein fibril)	2MXU	Homo sapiens
Aβ40 structures	Aβ40 monomer		2LNQ	Homo sapiens
EGFP	EGFP	Enhanced green fluorescent protein structure	2YOG	
Proteins used for peptide design	TTR	Transthyretin	P07309 (Uniprot)	Mus musculus
	Apo	Apolipoprotein A-II	P09813 (Uniprot)	Mus musculus
	LYZ	Lysozyme C	P61626 (Uniprot)	Homo sapiens
	CysC	Cystatin-C	P21460 (Uniprot)	Mus musculus
	SCARB1	Scavenger receptor class B member 1	Q61009 (Uniprot)	Mus musculus
	ACHA	Neuronal acetylcholine receptor subunit alpha-7	P49582 (Uniprot)	Mus musculus
	TGFB	Transforming growth factor beta-2 proprotein	P61812 (Uniprot)	Homo sapiens
	BPTF	Nucleosome-remodeling factor subunit BPTF	2F6J (incomplete structure)	Homo sapiens
Control peptide proteins	PrP	Major prion protein	P04925 (Uniprot)	Mus musculus
	NCAM1	Neural cell adhesion molecule 1	P13595 (Uniprot)	Mus musculus

Table S2. Protein origin and sequences used for further prediction and design. Marked are derived protein name, the whole aa length of the original protein and identification notes for the protein sequence FASTA. The whole aa sequence was shortened, as in this work the main focus was on the signal sequence located in the N-terminus of the protein. Yellow - sequences corresponding to original peptides for further modifications, green the PrP_{23-28} sequence added to the C-terminus of some peptides, underlined – predicted signal sequence, * – predicted cleavage site.

<table>
<tr><th>Derived peptide name</th><th>Protein length (aa)</th><th>Origin</th></tr>
<tr><td>PRNP</td><td>254</td><td>>sp|P04925|PRIO_MOUSE Major prion protein OS=Mus musculus OX=10090 GN=Prnp PE=1 SV=2</td></tr>
<tr><td colspan="3">MANLGYWLLALFVTMWTDVGLC*KKRPKPGGWNTGGSRYPGQGSPGGNRYPPQGGTWGQPHGGGWGQPHGGSWGQPHGGSWGQPHGGGWGQGGGTHNQWNKPSKPKTNLKHVAGAAAAGAVVGGLGG…</td></tr>
<tr><td>NCAM1</td><td>1115</td><td>>sp|P13595|NCAM1_MOUSE Neural cell adhesion molecule 1 OS=Mus musculus OX=10090 GN=Ncam1 PE=1 SV=3</td></tr>
<tr><td colspan="3">MLRTKDLIWTLFFLGTAVS*LQVDIVPSQGEISVGESKFFLCQVAGDAKDKDISWFSPNGEKLSPNQQRISVVWNDDDSSTLTIYNANIDDAGIYKCVVTAEDGTQSEATVNVKIFQKLMFKNAPTPQEFKEGEDAVIV…</td></tr>
<tr><td>TTR</td><td>147</td><td>sp|P07309|TTHY_MOUSE Transthyretin OS=Mus musculus OX=10090 GN=Ttr PE=1 SV=1</td></tr>
<tr><td colspan="3">MASLRLFLLCLAGLVFVSEA*GPAGAGESKCPLMVKVLDAVRGSPAVDVAVKVFKKTSEGSWEPFASGKTAESGELHGLTTDEKFVEGVYRVELDTKSYWKTLGISPFHEFADVVFTANDSGHRHYTIAALLSPYSYSTTAVVSNPQN</td></tr>
<tr><td>Apo</td><td>102</td><td>>sp|P09813|APOA2_MOUSE Apolipoprotein A-II OS=Mus musculus OX=10090 GN=Apoa2 PE=1 SV=2</td></tr>
<tr><td colspan="3">MKLLAMVALLVTICSLEG*ALVKRQADGPDMQSLFTQYFQSMTDYGKDLMEKAKTSEIQSQAKAYFEKTHEQLTPLVRSAGTSLVNFFSSLMNLEEKPAPAAK</td></tr>
<tr><td>LYZ</td><td>148</td><td>>sp|P61626|LYSC_HUMAN Lysozyme C OS=Homo sapiens OX=9606 GN=LYZ PE=1 SV=1</td></tr>
<tr><td colspan="3">MKALIVLGLVLLSVTVQG*KVFERCELARTLKRLGMDGYRGISLANWMCLAKWESGYNTRATNYNAGDRSTDYGIFQINSRYWCNDGKTPGAVNACHLSCSALLQDNIADAVACAKRVVRDPQGIRAWVAWRNRCQNRDVRQYVQGCGV</td></tr>
<tr><td>CysC</td><td>140</td><td>>sp|P21460|CYTC_MOUSE Cystatin-C OS=Mus musculus OX=10090 GN=Cst3 PE=1 SV=2</td></tr>
<tr><td colspan="3">MASPLRSLLFLLAVLAVAWA*ATPKQGPRMLGAPEEADANEEGVRRALDFAVSEYNKGSNDAYHSRAIQVVRARKQLVAGVNYFLDVEMGRTTCTKSQTNLTDCPFHDQPHLMRKALCSFQIYSVPWKGTHSLTKFSCKNA</td></tr>
<tr><td>SCARB1</td><td>509</td><td>>sp|Q61009|SCRB1_MOUSE Scavenger receptor class B member 1 OS=Mus musculus OX=10090 GN=Scarb1 PE=1 SV=1</td></tr>
</table>

MGGSSRARWVALGLGALGLLFAALGVVMILMVPSLIKQQVLKNVRIDPSSLSFGMWKEIPVPFYLSVYFFEVVNPNEVLN GQKPVVRERGPYVYREFRQKVNITFNDNDTVSFVENRSLHFQPDKSHGSESDYIV…		
ACHA	502	>sp|P49582|ACHA7_MOUSE Neuronal acetylcholine receptor subunit alpha-7 OS=Mus musculus OX=10090 GN=Chrna7 PE=1 SV=1
MCGRRGGIWLALAAALLHVSLQG*EFQRRLYKELVKNYNPLERPVANDSQPLTVYFSLSLLQIMDVDEKNQVLTTNIWL QMSWTDHYLQWNMSEYPGVKNVRFPDGQIWKPDILLYNSADERFDATFHTNVLVN…		
TGFB	414	>sp|P61812|TGFB2_HUMAN Transforming growth factor beta-2 proprotein OS=Homo sapiens OX=9606 GN=TGFB2 PE=1 SV=1
MHYCVLSAFLILHLVTVALS*LSTCSTLDMDQFMRKRIEAIRGQILSKLKLTSPPEDYPEPEEVPPEVISIYNSTRDLLQEKAS RRAAACERERSDEEYYAKEVYKIDMPPFFPSENAIPPTFYRPYFRIVRFDVSAMEKN…		
BPTF	3,046	>sp|Q12830|BPTF_HUMAN Nucleosome-remodeling factor subunit BPTF OS=Homo sapiens OX=9606 GN=BPTF PE=1 SV=3
MRGRRGRPPKQPAAPAAERCAPAPPPPPPPPTSGPIGGLRSRHRGSSRGRWAAAQAEVAPKTRLSSPRGGSSSRRKPPPPPP APPSTSAPGRGGRGGGGGRTGGGGGGGHLARTTAARRAVNKVVYDDHESEEE…		

Table S3. Prediction scores for signal sequences predicted with Signal-IP 5.0 [1], and PrediSi. For the CPP prediction two predictors were used: CellPPD [2], CPP predictor [3].

	Signal sequence prediction					CPP prediction from signal sequence (CellPPD and CPP predictor)
Protein	Cleavage site between aa/aa (if differs between predictors, then both marked)	Cleavage site probability (Signal-IP)	Signal peptide (PrediSi)	Signal peptide (Sec/SPI) (Signal-IP)	Other (Signal-IP)	
PRNP	22/23	0.8933	0.6853	0.9963	0.0037	Non-CPP
NCAM	19/20 or 22/23	0.5985	0.5003	0.9475	0.0525	Non-CPP
TTR	20/21	0.7597	0.9608	0.9986	0.0014	Non-CPP
Apo	18/19	0.6577	0.7133	0.9947	0.0053	Non-CPP
LYZ	18/19	0.9910	0.8696	0.9993	0.0007	Non-CPP
CysC	20/21	0.8385	1.0000	0.9922	0.0078	Non-CPP
SCARB1	- or 22/23	-	0.6784	0.0078	0.9922	Non-CPP
ACHA	23/24 or 22/23	0.6674	0.7771	0.9253	0.0747	Non-CPP
TGFB	20/21	0.3847	0.8021	0.8802	0.1198	Non-CPP
BPTF	-	-	0.0000	0.0043	0.9957	-
Aβ42, human	-	-	0.0000	0.0034	0.9966	-

Table S4. Summary of protein and peptide properties. For CPP prediction two main predictors were used (CellPPD [2], CPP predictor [3]), and additional CPP predictor, the MLCPP 2.0 [4]. Predictions of the therapeutic probability of peptide was predicted with CSM peptides [11]. nd – not determined.

	Protein´s implied effect on Aβ (↓/↑)	Includes signal sequence	Includes C-terminal $PrP_{23–28}$ (+/-)	Predicted CPP after modification/SVM score (CellPPD)	Prediction with MLCPP 2.0		Prediction with CSM peptides
					CPP/ probability	Uptake/ probability	Predicted over threshold >0.5
**PrP_{1-28}**	*n.d.*	+	+	CPP/1.00	CPP/0.99	Low/0.02	Anti-viral
**$NCAM1_{1–19}PrP_{23–28}$**		+	+	CPP/0.39	CPP/0.68	High/0.71	Anti-cancer, Anti-inflammatory
**$TTR_{1-21}PrP_{23–28}$**	↓	+	+	CPP/0.39	CPP/0.85	High/0.85	Anti-inflammatory, anti-viral
**$Apo_{1-19}PrP_{23–28}$**	↓	+	+	CPP/0.25	CPP/0.69	Low/0.03	Anti-cancer, anti-inflammatory, Anti-viral, CPP
**$LYZ_{1-24}PrP_{23–28}$**	↓	+	+	CPP/0.33	CPP/0.55	Low/0.06	Anti-cancer, anti-inflammatory, Anti-viral
**$LYZ_{1-19}PrP_{23–28}$**	↓	+	+	CPP/0.31	nd	nd	Anti-cancer, anti-inflammatory, Anti-viral, CPP
**pCysC**	↓/↑	+	-	CPP/0.09	Non-CPP/0.51	-	Anti-inflammatory, Anti-viral, CPP
**pSCB**	↓	+/-	-	CPP/0.07	CPP/0.94	Low/0.0008	Anti-bacterial, anti-inflammatory, anti-viral
**pACHA**	↑	+	-	CPP/0.08	CPP/0.95	High/0.66	Anti-cancer, anti-inflammatory, anti-viral, quorum sensing
**$TGFB_{25-50}$**	↑	+ (protein)/ - (peptide)	-	Non-CPP/-0.12	Non-CPP/0.01	-	Anti-cancer, anti-inflammatory, anti-viral
**$BPTF_{1-19}$**	0	-	-	CPP/0.22	CPP/0.83	High/0.99	Anti-inflammatory

Table S5. Calculated peptide properties using PPC ver 3.1. a - Solubility calculated with CamSol v2.2. Scores below -1 is aggregation promoting, above 1 solubility promoting. [5,6]. *UPLC analysis was performed on purified peptides using water/acetonitrile gradient (supplemented with 0.1% TFA) with AcN 5 – 95% (13 min).

				**Amino acid composition (rounded %)**					
**Peptide**	pI (pH)	Mw (g/mol)	Net charge at pH 7	Acidic	Basic	Neutral	Hydro-phobic	Solubility[a]	Retention time (min) in UPLC*
**PrP_{1-28}**	10.54	3252	3.9	3.6	14.3	25	57	-1.068007	9.099
**$NCAM1_{1–19}$-$PrP_{23–28}$**	12.2	2947	6	4	24	28	44	0.718149	6.366
**$TTR_{1-21}PrP_{23–28}$**	11.49	2945	4.9	3.7	18.5	22.2	55.6	0.637992	9.319
**Apo_{1-19}-PrP_{23-28}**	11.1	2711	4.9	4	20	20	56	0.737654	8.743
**$LYZ_{1-24}PrP_{23-28}$**	11.63	3351	6.9	3.3	23.3	23.3	50	0.208654	8.610
**pCysC**	14	3234	6	0	16.7	26.7	56.7	0.431699	9.992
**pSCB**	14	2758	6	0	18.5	25.9	55.6	1.421306	7.723
**pACHA**	12.58	2875	6	0	23.1	23.1	53.9	0.865362	7.847
**pTGFB**	11.39	3091	4	11.5	23.1	23.1	42.3	1.948518	6.089
**pBPTF**	12.71	2102	6	5.3	31.6	36.9	26.3	3.027363	2.491

Table S6. Predicted secondary structures for the peptides.

Secondary structure prediction was done with predictors, and based on the peptide sequence of modified peptide shown in Table 1. Predictor 1, 2 and 3 were used to predict probable probable alpha helix, beta strand, turn, coil from peptide sequences. [5-9]. CF – Chou-Fasman prediction. NN – neural network prediction. For P2 the Joint includes CF, GOR-I, and NN prediction.

Predictor	Sequence
	**PrP$_{1-28}$**
Amphiphilicity Helix[P1]	MANLGYWLLALFVTMWTDVGLCKKRPKP
CF prediction[P1]	MANLGYWLLALFVTMWTDVGLCKKRPKP
GOR-I prediction[P1]	MANLGYWLLALFVTMWTDVGLCKKRPKP
Joint prediction[P2]	MANLGYWLLALFVTMWTDVGLCKKRPKP
s2Dv2[P3]	MANLGYWLLALFVTMWTDVGLCKKRPKP
	**NCAM1$_{1–19}$PrP$_{23–28}$**
Amphiphilicity Helix[P1]	MLRTKDLIWTLFFLGTAVSKKRPKP
CF prediction[P1]	MLRTKDLIWTLFFLGTAVSKKRPKP
GOR-I prediction[P1]	MLRTKDLIWTLFFLGTAVSKKRPKP
Joint prediction[P2]	MLRTKDLIWTLFFLGTAVSKKRPKP
s2Dv2[P3]	MLRTKDLIWTLFFLGTAVSKKRPKP
	**TTR$_{1-21}$PrP$_{23–28}$**
Amphiphilicity Helix[P1]	MASLRLFLLCLAGLVFVSEAGKKRPKP
CF prediction[P1]	MASLRLFLLCLAGLVFVSEAGKKRPKP
GOR-I prediction[P1]	MASLRLFLLCLAGLVFVSEAGKKRPKP
Joint prediction[P2]	MASLRLFLLCLAGLVFVSEAGKKRPKP
s2Dv2[P3]	MASLRLFLLCLAGLVFVSEAGKKRPKP
	**Apo$_{1-19}$PrP$_{23–28}$**
Amphiphilicity Helix[P1]	MKLLAMVALLVTICSLEGAKKRPKP
CF prediction[P1]	MKLLAMVALLVTICSLEGAKKRPKP
GOR-I prediction[P1]	MKLLAMVALLVTICSLEGAKKRPKP
Joint prediction[P2]	MKLLAMVALLVTICSLEGAKKRPKP
s2Dv2[P3]	MKLLAMVALLVTICSLEGAKKRPKP
	**LYZ$_{1-24}$PrP$_{23–28}$**
Amphiphilicity Helix[P1]	MKALIVLGLVLLSVTVQGKVFERCKKRPKP
CF prediction[P1]	MKALIVLGLVLLSVTVQGKVFERCKKRPKP
GOR-I prediction[P1]	MKALIVLGLVLLSVTVQGKVFERCKKRPKP
Joint prediction[P2]	MKALIVLGLVLLSVTVQGKVFERCKKRPKP
s2Dv2[P3]	MKALIVLGLVLLSVTVQGKVFERCKKRPKP
	**LYZ$_{1-19}$PrP$_{23–28}$**
Amphiphilicity Helix[P1]	MKALIVLGLVLLSVTVQGKKKRPKP
CF prediction[P1]	MKALIVLGLVLLSVTVQGKKKRPKP
GOR-I prediction[P1]	MKALIVLGLVLLSVTVQGKKKRPKP
Joint prediction[P2]	MKALIVLGLVLLSVTVQGKKKRPKP
s2Dv2[P3]	MKALIVLGLVLLSVTVQGKKKRPKP
	**pCysC**
Amphiphilicity Helix[P1]	MASPLRSLLFLLAVLAVAWAATPKQGPRKK
CF prediction[P1]	MASPLRSLLFLLAVLAVAWAATPKQGPRKK
GOR-I prediction[P1]	MASPLRSLLFLLAVLAVAWAATPKQGPRKK
Joint prediction[P2]	MASPLRSLLFLLAVLAVAWAATPKQGPRKK
s2Dv2[P3]	MASPLRSLLFLLAVLAVAWAATPKQGPRKK
	**pSCB**
Amphiphilicity Helix[P1]	MGGSSRARWVALGLGALGLLFAAKKRA
CF prediction[P1]	MGGSSRARWVALGLGALGLLFAAKKRA

GOR-I prediction[P1]	MGGSSRARWVALGLGALGLLFAAKKRA
Joint prediction[P2]	MGGSSRARWVALGLGALGLLFAAKKRA
s2Dv2[P3]	MGGSSRARWVALGLGALGLLFAAKKRA
**pACHA**	
Amphiphilicity Helix[P1]	MCGRRGGIWLALAAALLHVSLQRRPK
CF prediction[P1]	MCGRRGGIWLALAAALLHVSLQRRPK
GOR-I prediction[P1]	MCGRRGGIWLALAAALLHVSLQRRPK
Joint prediction[P2]	MCGRRGGIWLALAAALLHVSLQRRPK
s2Dv2[P3]	MCGRRGGIWLALAAALLHVSLQRRPK
**TGFB$_{25-50}$**	
Amphiphilicity Helix[P1]	STLDMDQFMRKRIEAIRGQILSKLKL
CF prediction[P1]	STLDMDQFMRKRIEAIRGQILSKLKL
GOR-I prediction[P1]	STLDMDQFMRKRIEAIRGQILSKLKL
Joint prediction[P2]	STLDMDQFMRKRIEAIRGQILSKLKL
s2Dv2[P3]	STLDMDQFMRKRIEAIRGQILSKLKL
**BPTF$_{1-19}$**	
Amphiphilicity Helix[P1]	MRGRRGRPPKQPAAPAAER
CF prediction[P1]	MRGRRGRPPKQPAAPAAER
GOR-I prediction[P1]	MRGRRGRPPKQPAAPAAER
Joint prediction[P2]	MRGRRGRPPKQPAAPAAER
s2Dv2[P3]	MRGRRGRPPKQPAAPAAER

Table S7. Additional predictions for peptides used in this work.

**Peptide**	**Toxin peptide prediction (ToxinPred)**	**Allergen (AllerTop, AllergenFP)**	**Hemolysis probability predictor (HemoPI) (0 – non-hemolytic, 1 – hemolytic) SVM (HemoPI-2) based**	**Anticancer peptide prediction (AntiCP)**	**BBB penetrating peptide prediction (BBPpredict) Y/N and probability 0-1**
**PrP$_{1-28}$**	Non-toxin	Probable non-allergen	0.5	Non-antiCP	No, 0.41
**NCAM1$_{1-19}$-PrP$_{23-28}$**	Non-toxin	Probable allergen	0.55	Non-antiCP	Yes, 0.66
**TTR$_{1-21}$PrP$_{23-28}$**	Non-toxin	Probable allergen	0.61	Non-antiCP	No, 0.25
**Apo$_{1-19}$-PrP$_{23-28}$**	Non-toxin	Probable non-allergen	0.52	AntiCP	Yes, 0.55
**LYZ$_{1-24}$PrP$_{23-28}$**	Non-toxin	Probable non-allergen	0.51	Non-antiCP	Yes, 0.51
**pCysC**	Non-toxin	Probable non-allergen	0.51	AntiCP	No, 0.33
**pSCB**	Non-toxin	Probable allergen	0.51	AntiCP	Yes, 0.55
**pACHA**	Non-toxin	Probable non-allergen	0.55	AntiCP	Yes, 0.73
**pTGFB**	Non-toxin	Probable non-allergen	0.5	Non-antiCP	Yes, 0.52
**pBPTF**	Non-toxin	Probable non-allergen	0.49	Non-antiCP	Yes, 0.66

Table S8. Additional peptides used in this work, their sequences, and source.

Name	Peptide sequence	Reference
Aβ42, human	DAEFRHDSGYEVHHQKLVFFAEDVGSNKGAIIGLMVGGVVIA	GeneScript, 107761-42-2; AnaSpec, AS-72216
Aβ40, human	DAEFRHDSGYEVHHQKLVFFAEDVGSNKGAIIGLMVGGVV	SensoLyte kit component, AS-72213
NickFect51	aAGYLLGObINLKALAALAKKIL-NH2	[17]

a – Fatty acid residue, stearoyl

b – synthesis is continued from the side-chain amino group instead of alpha-amino group

Supplementary Figures:

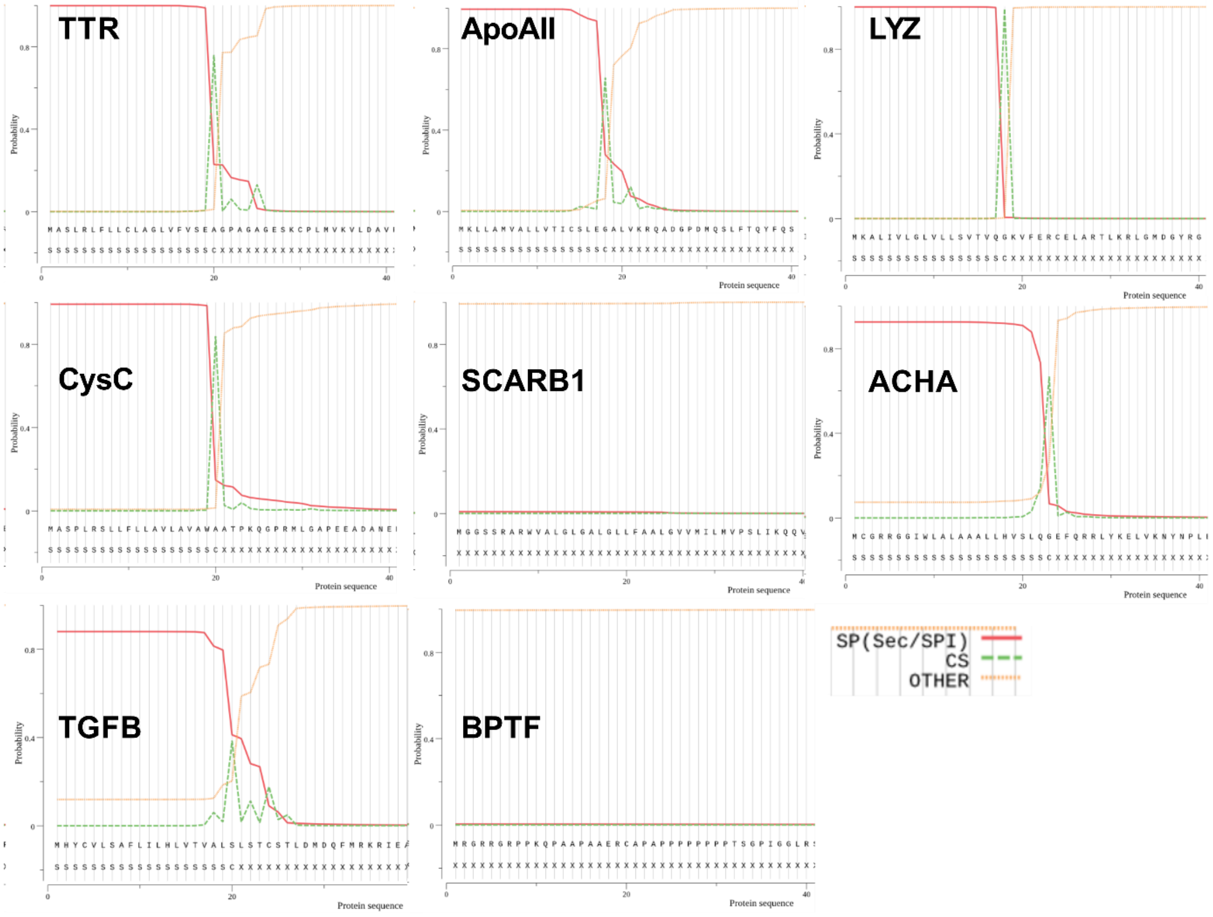

Figure S1. Signal sequences and cleavage sites predicted from protein sequences. For prediction Signal-IP 5.0 was used [1]. SP – secreted, CS – cleavage site. Only the first part of the protein N-terminal sequence was included in the prediction due to size limits.

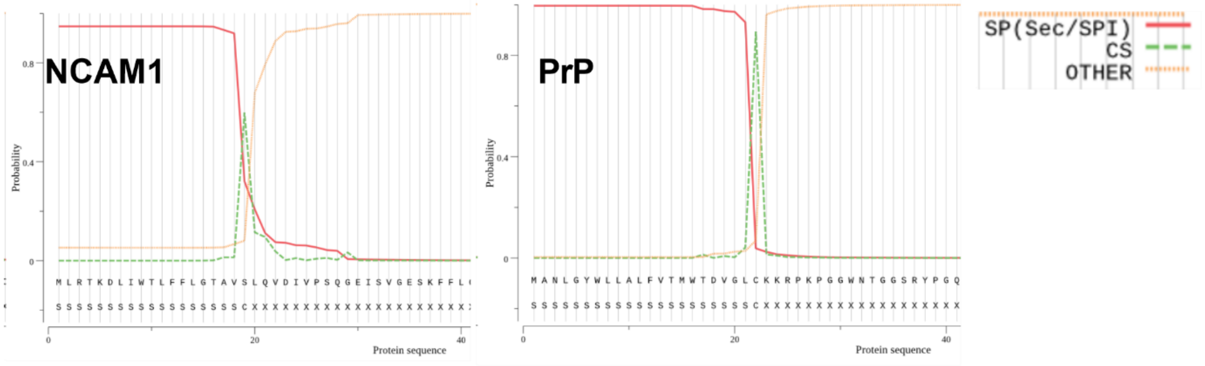

Figure S2. Signal sequences and cleavage sites predicted from protein sequences from which the control peptides are derived from. For prediction Signal-IP 5.0 was used [1]

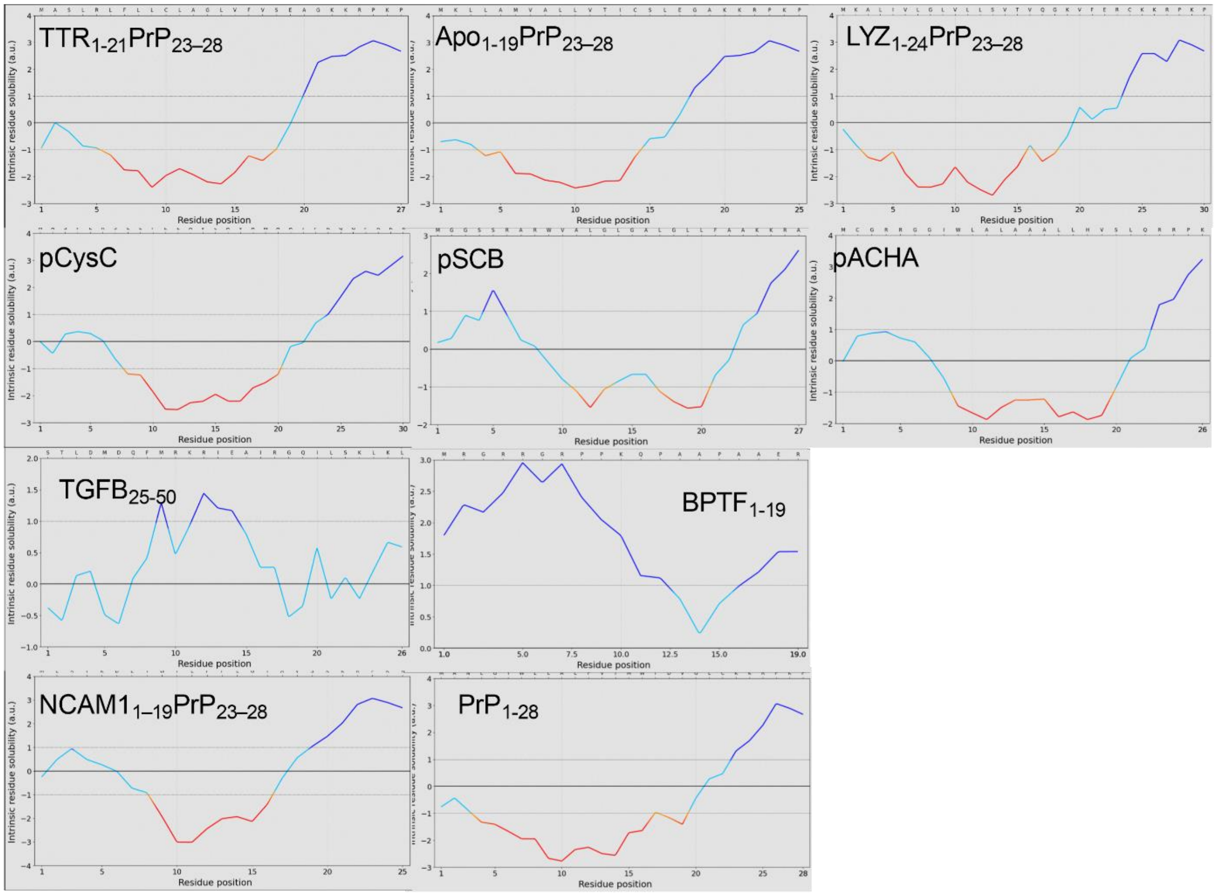

Figure S3. Solubility profiles predicted from designed peptides and control peptides. For prediction. CamSol v2.2 was used [5,6]. On the image with the intrinsic solubility profile, below -1 is aggregation promoting, above 1 solubility promoting.

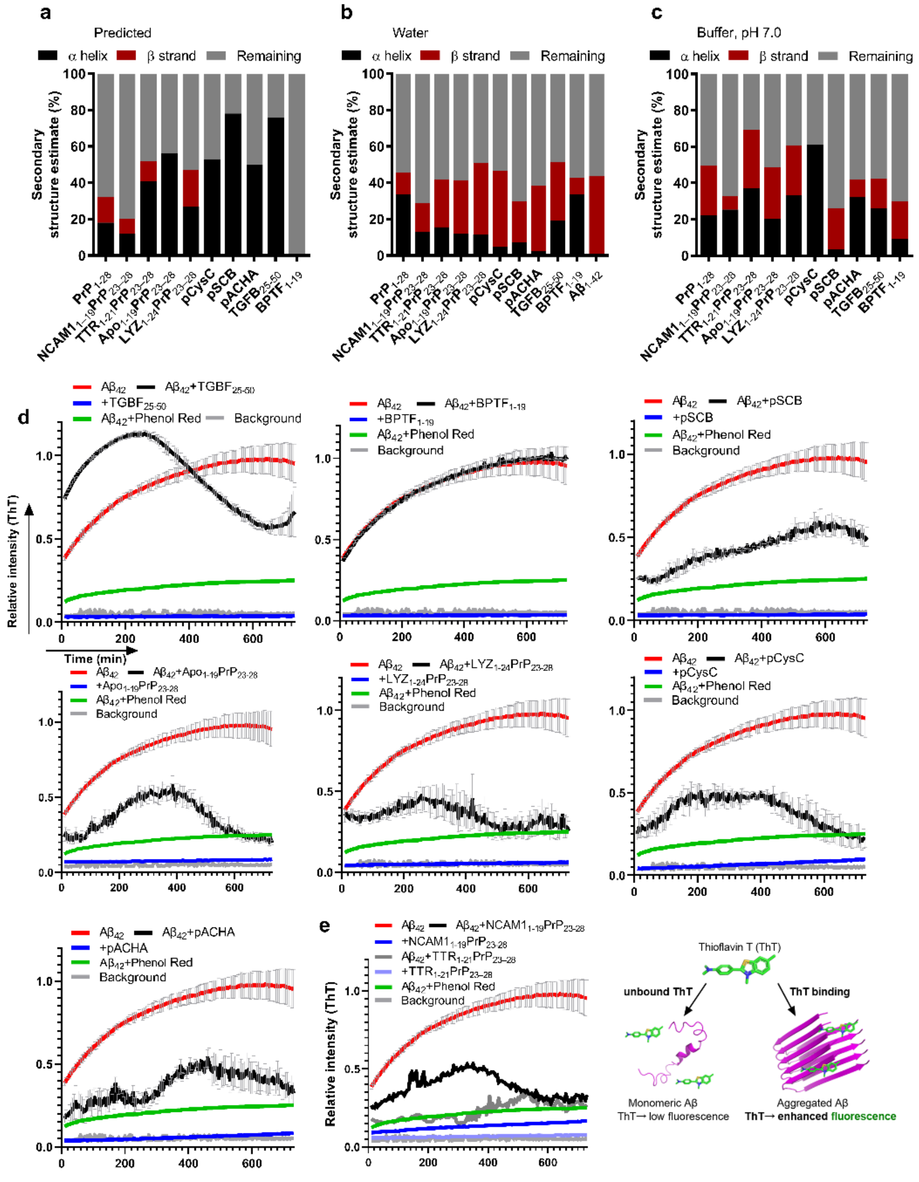

Figure S4. **Secondary structure of the peptides and effect of Aβ42**. a) Secondary structure of the peptides as predicted with s2Dv2, derived from CD spectra in b) water or c) buffer and calculated with BeStSel [10]. d) Reduction of $A\beta_{1\text{-}42}$ peptide aggregation in the presence of peptides. e) Comparison of peptides shown in Figure 2, with controls.

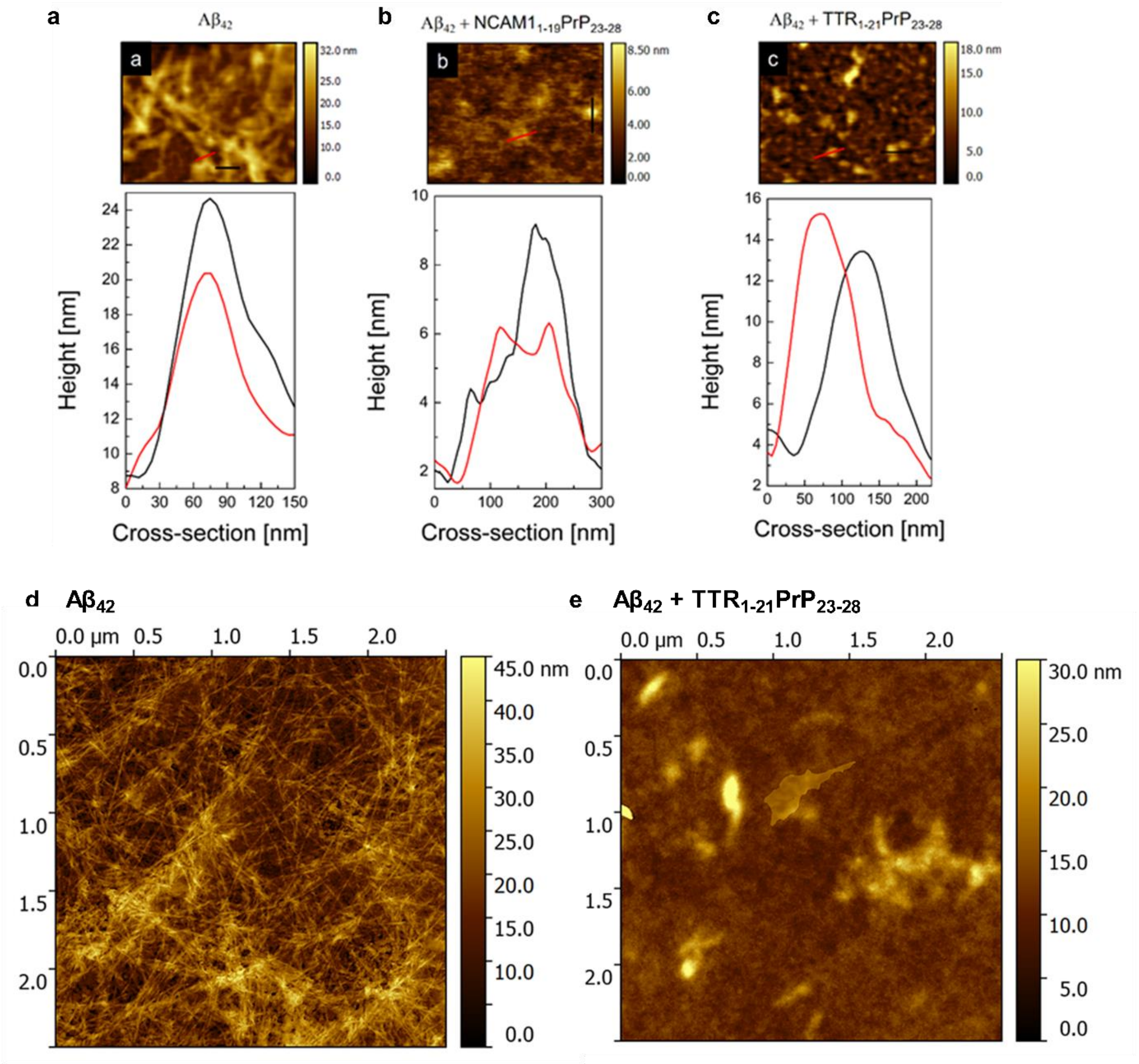

Figure S5: Atomic force microscopy data from samples containing (a) the $A\beta_{42}$ peptide, and mixtures with (b) the $NCAM1_{1-19}PrP_{23-28}$ and (c) the $TTR_{1-21}PrP_{23-28}$ inhibitors. On the bottom row, it is shown cross-section profiles from the zones indicated in the topography images. (d) High resolution AFM topography image from $A\beta_{42}$ fibers showing in detail the intricate network of amyloid structures. E) High-resolution AFM image from a sample containing $A\beta_{42}$ + $TTR_{1-21}PrP_{23-28}$ peptides showing the formation of amorphous film populated with irregular aggregates.

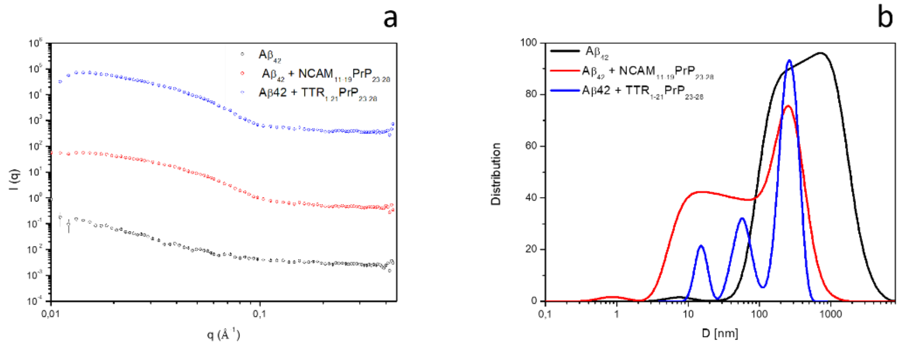

Figure S6: (a) small-angle scattering curves from solutions containing Aβ42 aggregates either in the absence (black) or in the presence of the amyloid inhibitors. (b) dynamic light scattering data showing size distributions of particles.

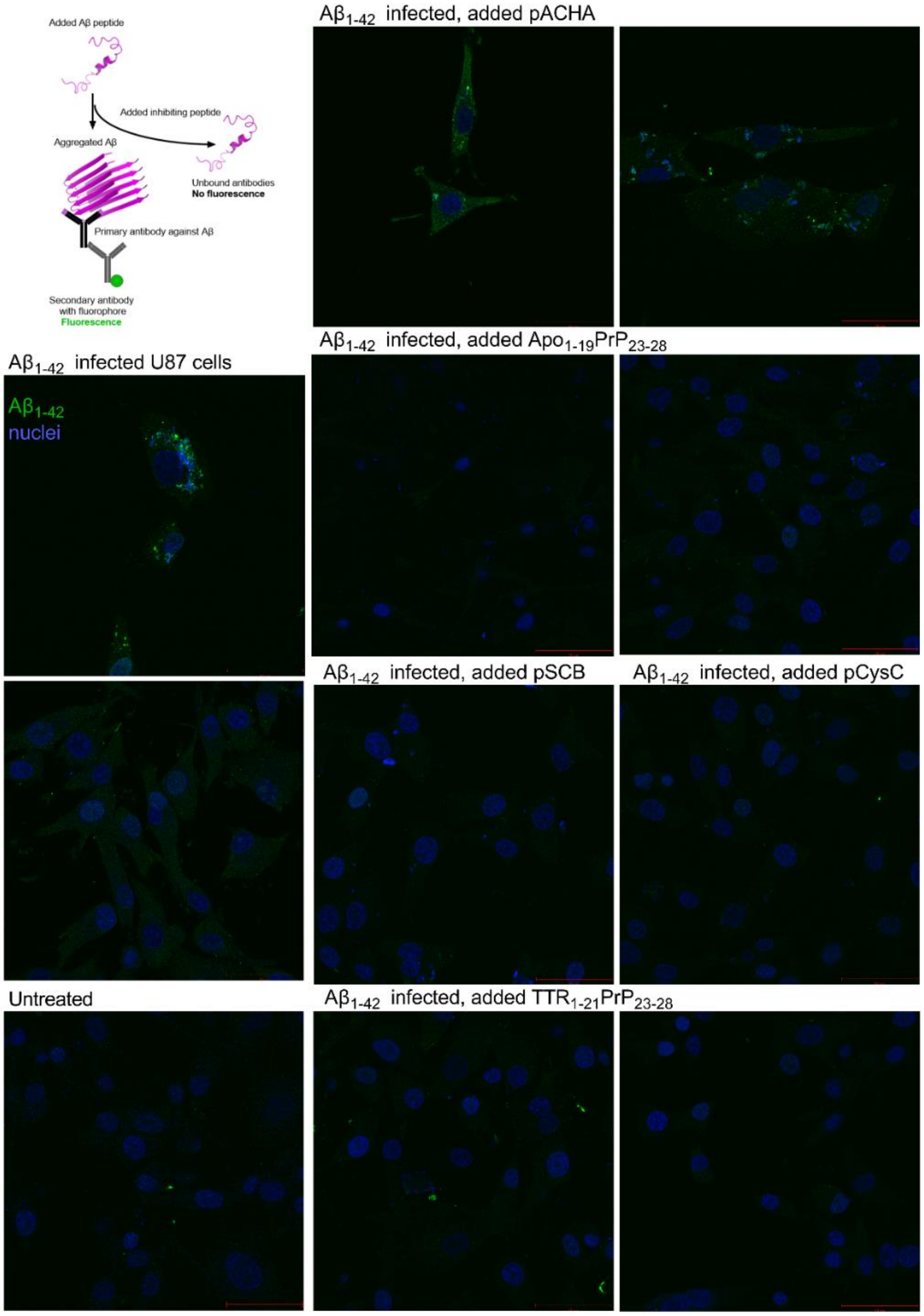

Figure S7. Reduction of Aβ1-42 peptide aggregates on cells. Amyloid aggregates labelled with antibody against, secondary antibody with 488 fluorophore. Nuclei = DAPI. 63 x magnification. U87 cells infected with Aβ1-42 peptide (5 µM) and aggregates. Peptides at 5 µM final concentration, co-incubation 48 h. IHC primary antibody against Aβ1-42, secondary 488 fluorescent label, DAPI for visualizing nuclei Reduction of aggregation in U87 cells. IHC. Amyloid peptide added to cells. AAP peptides added to cells. 24 h later the cells are fixed, marked with primary antibody against AB1-42 and secondary 488 fluorophore bearing antibody. Nuclei stained with DAPI.

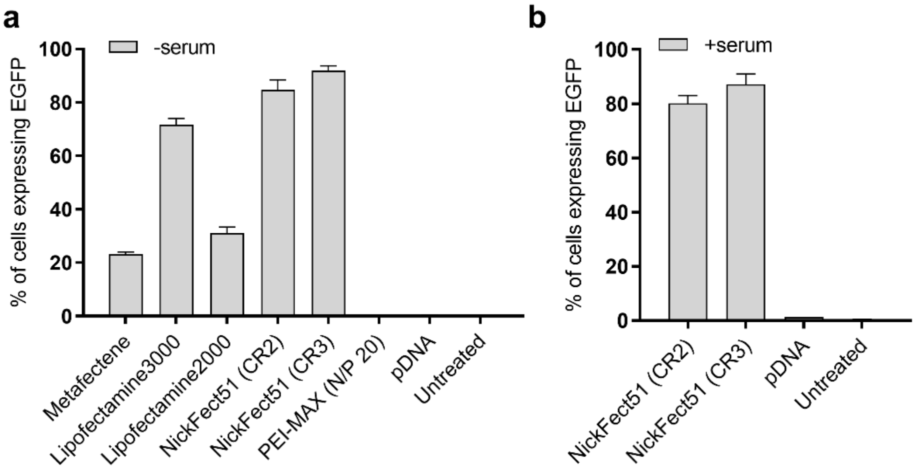

Figure S8. Transfected U87 cell population 24 h post-transfection. 48 h prior transfection 60,000 cells per well were seeded on a 24 well plate. 0.5 µg of green fluorescent protein expressing plasmid (pEGFP-C1) was transfected per well and 24 h post-transfection the cell population with signal from expressed GFP was determined using flow cytometry. As threshold for signal ~1% of untreated cell population was set as GFP+. Commercial treansfection reagents Metafectene, Lipofectamine3000 (LF3000) and Lipofectamine2000 (LF2000) were used according to manufacturer´s instructions. For PEI-MAX N/P 30 was used for complex formation between the reagent and pDNA. Cell-penetraring peptide NickFect51 was mixed with pDNA according to charge ratio (CR – ratio of peptide positive net charges and pDNA negatively charged backbone) with excess of peptide.

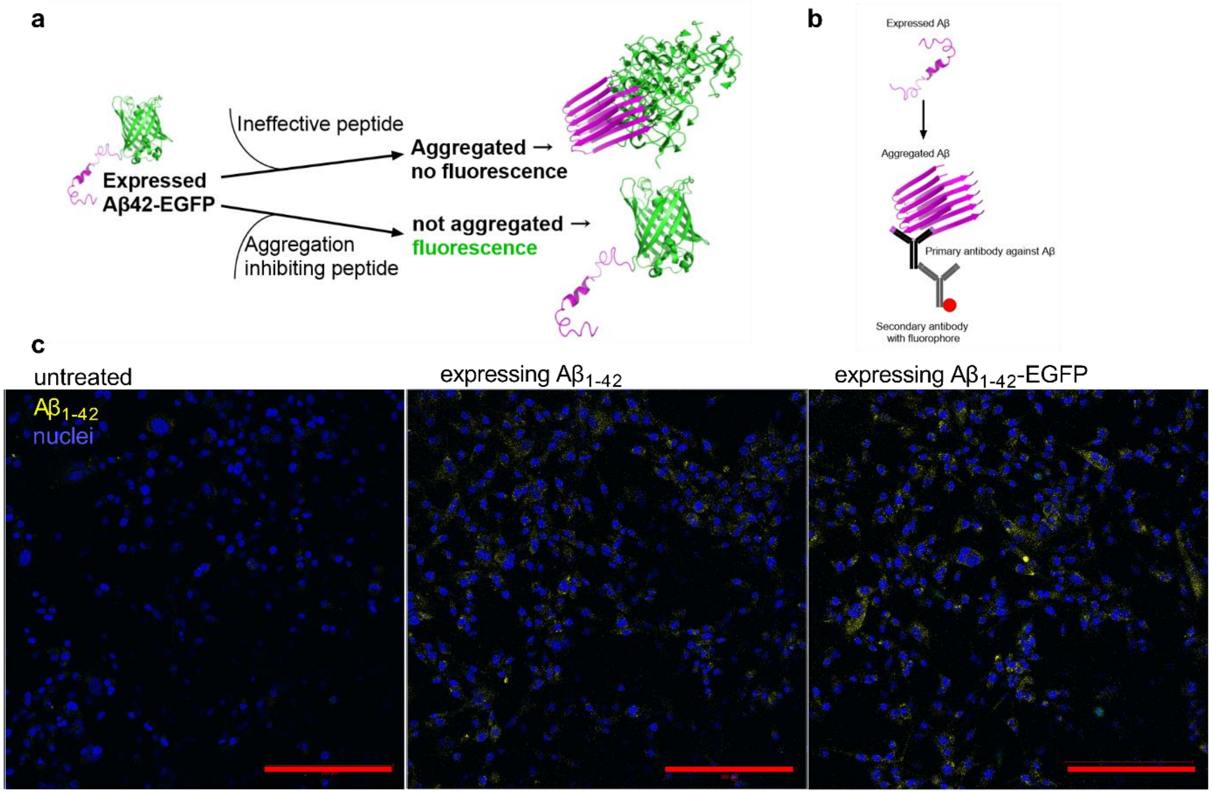

Figure S9. A$\beta_{1\text{-}42}$ detected from U87 cells transfected with plasmid expressing A$\beta_{1\text{-}42}$ or A$\beta_{1\text{-}42}$-GFP. a) Generalized scheme of aggregation assay principle. b) Scheme of immunohistochemistry against Aβ_{42} aggregates. c) Antibody labelled Aβ_{42} after transfection and expression of A$\beta_{1\text{-}42}$-EGFP or A$\beta_{1\text{-}42}$ from plasmid in U87 cells. 24 h post transfection cells were fixed and primary antibody against A$\beta_{1\text{-}42}$ was used to detect aggregates, and secondary antibody with AF568 fluorescent label (pseudo-colorized to yellow) was used to label the primary antibodies bound to aggregates. For visualizing nuclei DAPI was used. Images taken with 20 x objective. Red bar marks 200 µm.

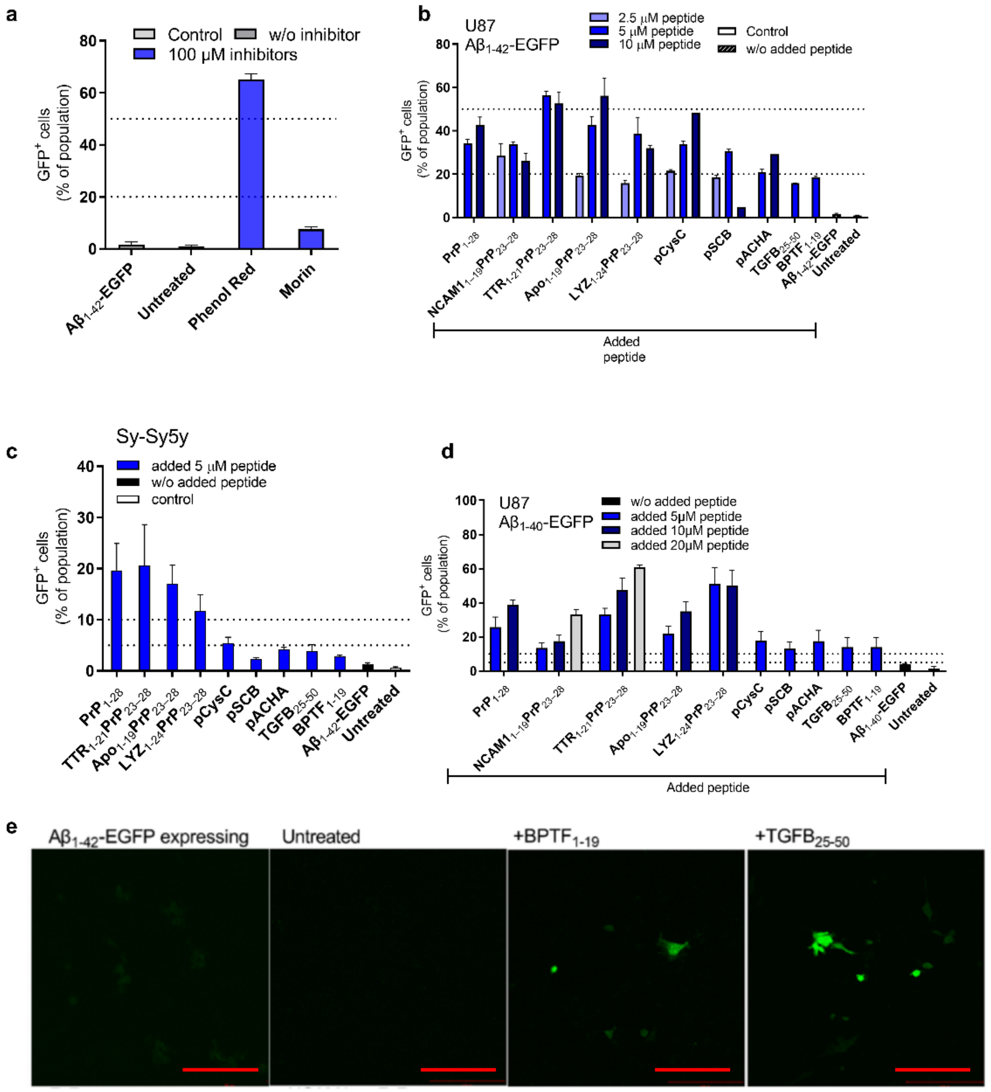

Figure S10. Reduction of amyloid aggregation with transfected SH-Sy5y and U87 cells, assessed by flow cytometry 24 h post-addition of peptides. Transfection of 0.5 µg Aβ42-GFP fusion protein expressing plasmid with NF51. a) Control inhibitors Phenol Red and Morin used to reduce $A\beta_{1-42}$-EGFP aggregation on U87 cells. $A\beta_{1-42}$-EGFP aggregation reduction with peptides added to U87 b) or Sy-Sy5y (c) cells transfected with 0.5 µg Aβ42-GFP fusion protein expressing plasmid. After 2 h AAP peptides were added to the cells with shown final concentration on cells. 24 h after addition of peptides, cells were detached and population of fluorescent positive cells were detected by flow cytometry. d) Reduction of $A\beta_{1-40}$-EGFP aggregation with peptides assessed by flow cytometry to determine GFP+ cell population. e) Confocal microscopy live cell images of controls and peptide treated groups. After transfection media was replaced with fresh media and peptides were added at the final concentration of 10 µM. 24 h post-addition of peptides images were taken to detect fluorescent signal from expressed $A\beta_{42}$-EGFP. Red bar 200 µm. Images taken with 63 x, oil immersion objective.

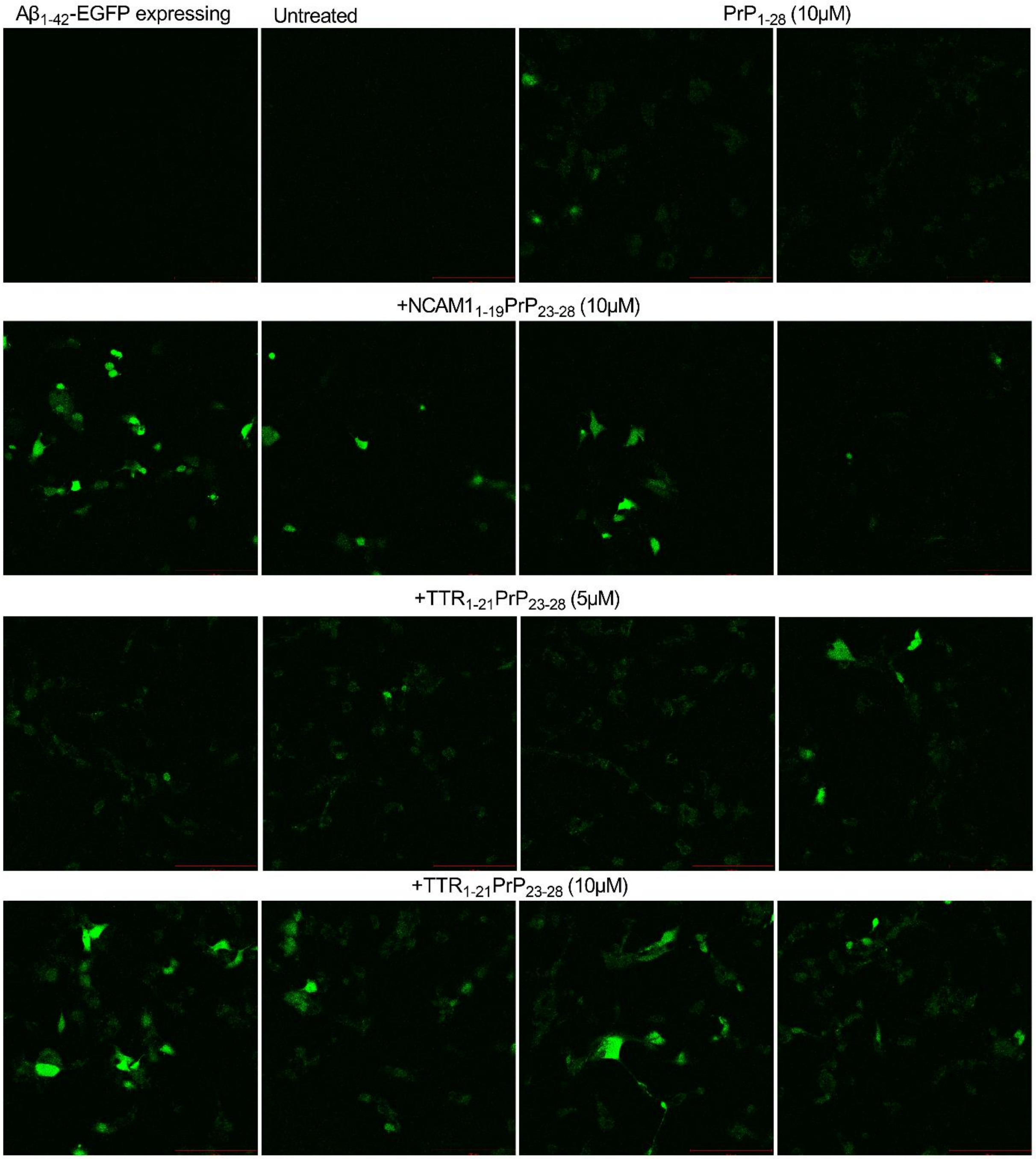

Figure S11. Confocal images of U87 cells transfected with plasmid expressing Aβ$_{1-42}$-EGFP and treated with peptides. Reduction expressed of Aβ aggregation assessed by confocal microscopy. U87 cells transfected with 0.5 µg Aβ$_{1-42}$-EGFP fusion protein expressing plasmid. After 2 h AAP peptides were added to the cells with the shown final concentration on cells. 24 h after addition of peptides confocal images were taken with confocal microscope. 63 x oil immersion objective. Band 200 µm.

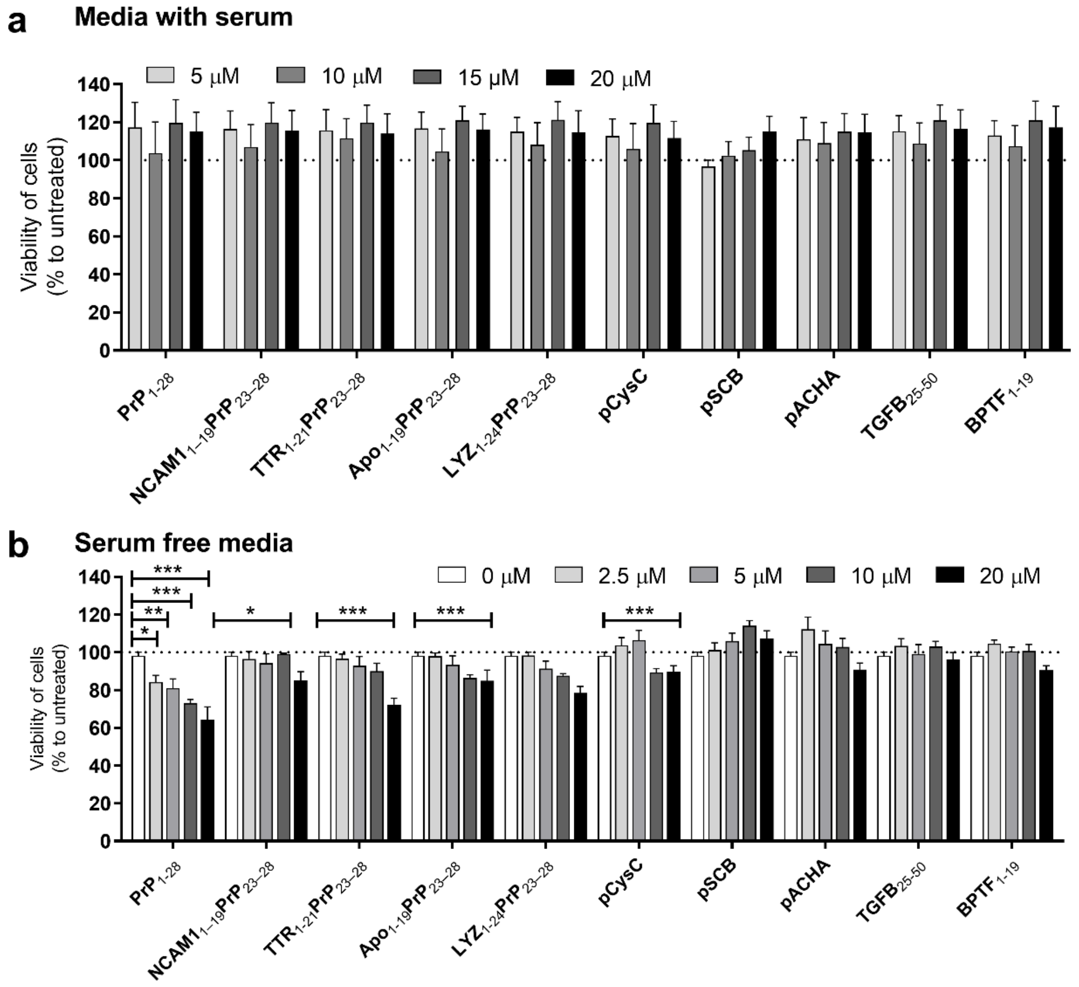

Figure S12. Assessment of cell viability after addition of peptides, by using MTS based toxicity assay. U87 cells were seeded 24 h prior experiment. On experiment day media was replaced with serum containing a) or serum free b) media and peptides added with shown final concentrations. 21 h post-addition of peptides detection reagent was added and incubated further for 3 h. Then absorbance was detected and results converted to percentage to untreated cells (100%).

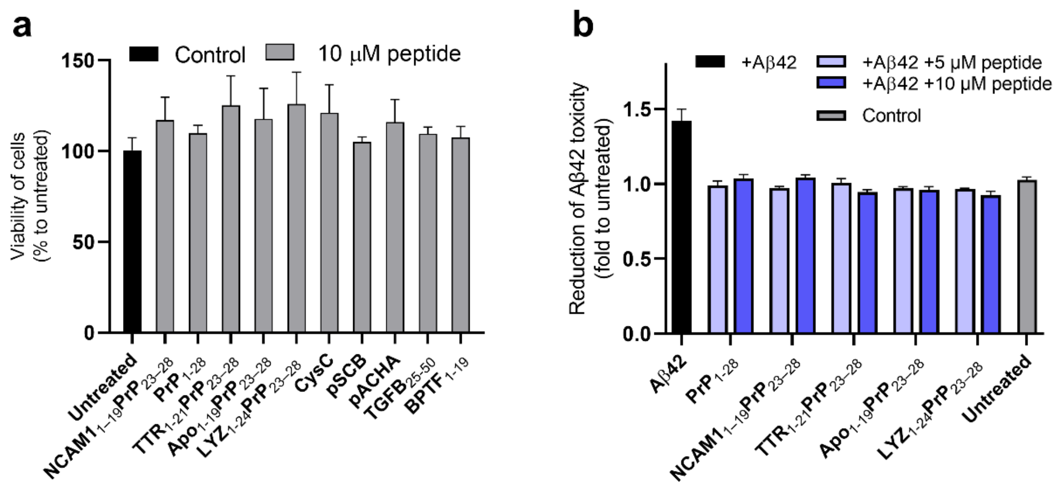

Figure S13. Viability of U87 cells and reduction of amyloid toxicity assessed with ToxGreen assay. U87 cells were seeded on a black 96 well plate one-day prior experiment. On experiment day media was replaced with fresh serum containing media and a) peptides were added to the cells or b) 10 µM Aβ_{42} peptide was added to the cells and then peptides added at the shown final concentrations. After 24 h incubation ToxGreen reagent was added to detect dead cells and fluorescence was measured from each well. In a) the fluorescence was normalized to untreated cells and shown as percentage to untreated. In b) the results are shown as fluorescence fold change to untreated cells. Then the reduction of fluorescence (reduction of cell death) was detected in groups where peptides were added.

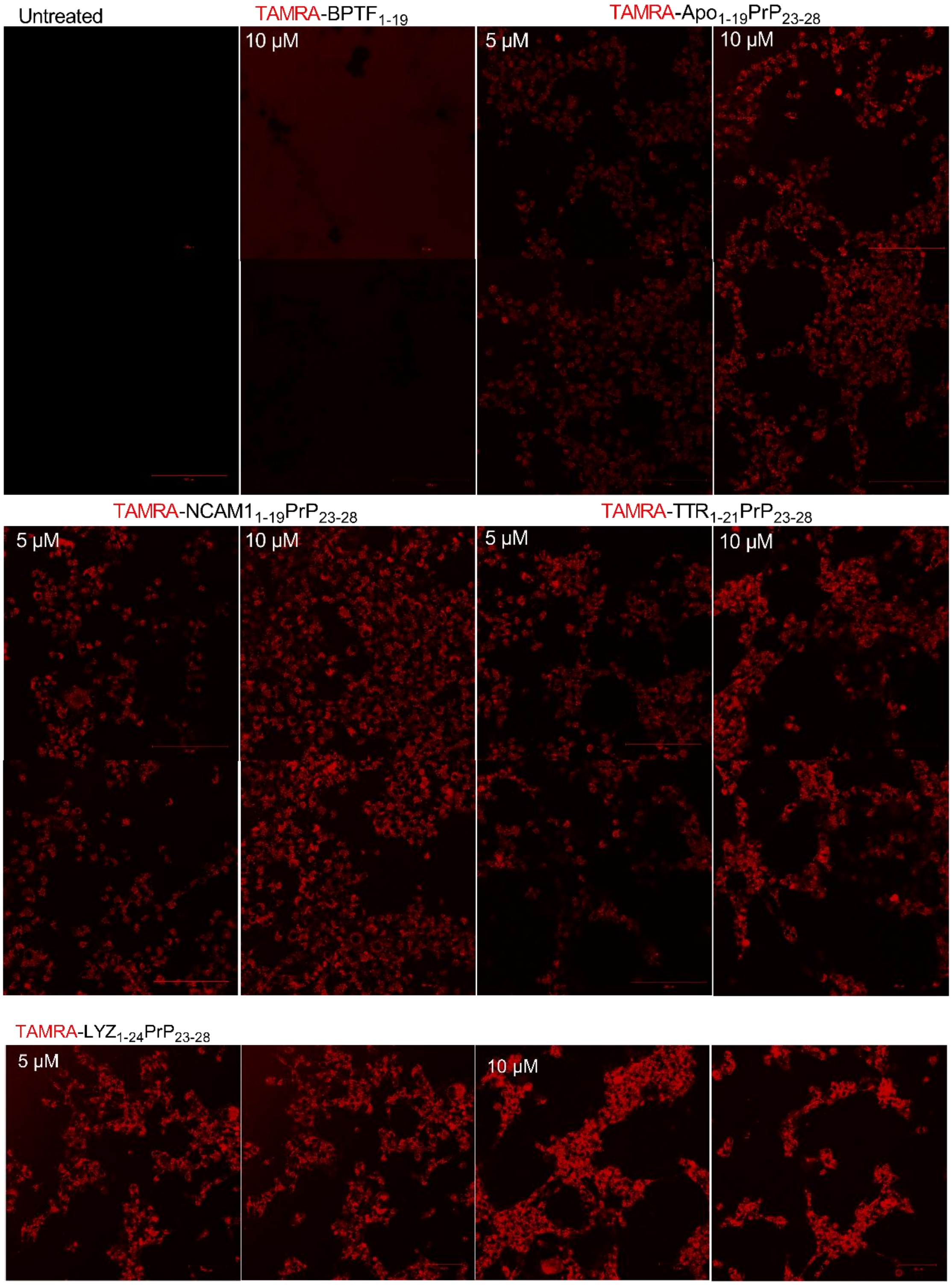

Figure S14. Internalization of fluorescently labelled peptides into U87 cells. U87 cells treated with shown final concentrations of fluorescently labelled peptides and incubated for 2-4 h, following live cell imaging with confocal microscope. 20 x objective, Red bar 200 μm.

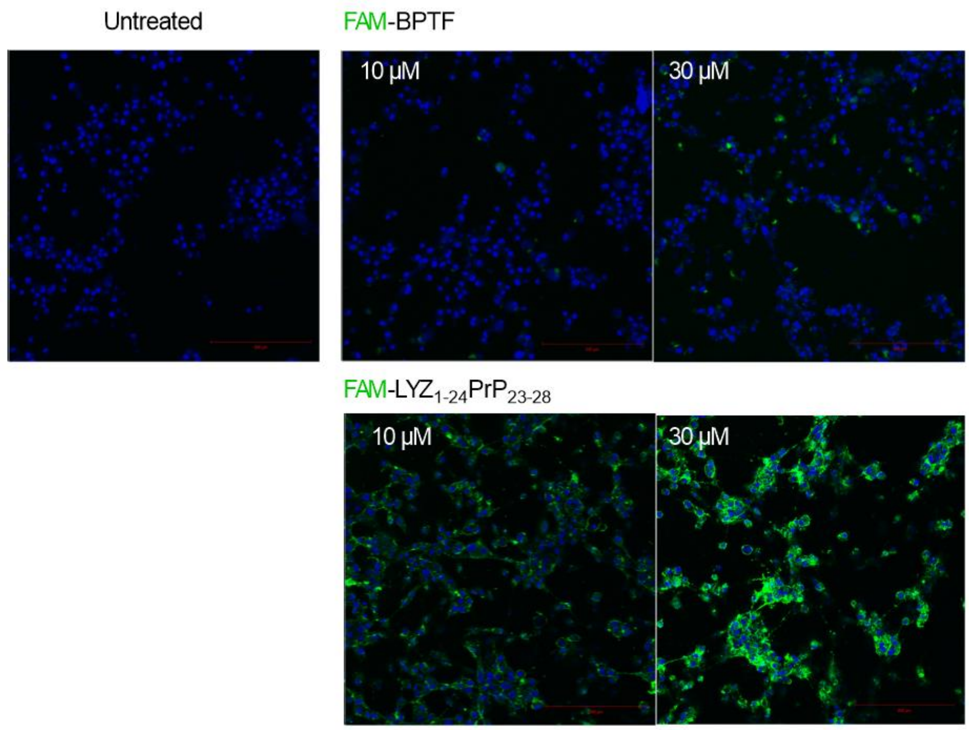

Figure S15. Internalization of fluorescently labelled peptides into U87 cells. U87 cells treated with shown final concentrations of fluorescently labelled peptides and incubated for 2-4 h, following live cell imaging with confocal microscope. 20 x objective, Red bar 200 µm.

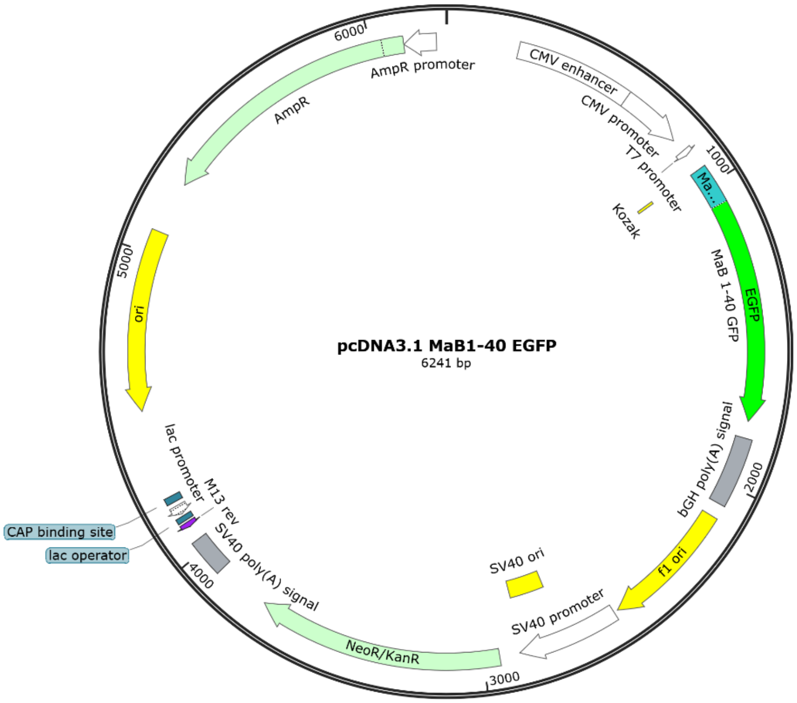

Figure S16. Plasmid construct exemplified by Aβ40-EGFP fusion protein expressing pcDNA3.1 plasmid.
